# A-Lister: a tool for analysis of differentially expressed omics entities across multiple pairwise comparisons

**DOI:** 10.1186/s12859-019-3121-x

**Published:** 2019-11-19

**Authors:** Stanislav A. Listopad, Trina M. Norden-Krichmar

**Affiliations:** 1 Department of Computer Science, University of California Irvine, California, USA; 2 Department of Epidemiology, University of California Irvine, California, USA

**Keywords:** Differential expression analysis, Differentially expressed genes (DEGs), Differentially expressed proteins (DEPs), Differentially methylated regions/positions (DMRs/DMPs), Python

## Abstract

**Background:**

Researchers commonly analyze lists of differentially expressed entities (DEEs), such as differentially expressed genes (DEGs), differentially expressed proteins (DEPs), and differentially methylated positions/regions (DMPs/DMRs), across multiple pairwise comparisons. Large biological studies can involve multiple conditions, tissues, and timepoints that result in dozens of pairwise comparisons. Manually filtering and comparing lists of DEEs across multiple pairwise comparisons, typically done by writing custom code, is a cumbersome task that can be streamlined and standardized.

**Results:**

A-Lister is a lightweight command line and graphical user interface tool written in Python. It can be executed in a differential expression mode or generic name list mode. In differential expression mode, A-Lister accepts as input delimited text files that are output by differential expression tools such as DESeq2, edgeR, Cuffdiff, and limma. To allow for the most flexibility in input ID types, to avoid database installation requirements, and to allow for secure offline use, A-Lister does not validate or impose restrictions on entity ID names. Users can specify thresholds to filter the input file(s) by column(s) such as *p*-value, q-value, and fold change. Additionally, users can filter the pairwise comparisons within the input files by fold change direction (sign). Queries composed of intersection, fuzzy intersection, difference, and union set operations can also be performed on any number of pairwise comparisons. Thus, the user can filter and compare any number of pairwise comparisons within a single A-Lister differential expression command.

In generic name list mode, A-Lister accepts delimited text files containing lists of names as input. Queries composed of intersection, fuzzy intersection, difference, and union set operations can then be performed across these lists of names.

**Conclusions:**

A-Lister is a flexible tool that enables the user to rapidly narrow down large lists of DEEs to a small number of most significant entities. These entities can then be further analyzed using visualization, pathway analysis, and other bioinformatics tools.

## Background

With the recent explosion of genomic data, researchers are storing, cleaning, processing, and analyzing increasingly large volumes of data [1]. Differential expression studies account for a large portion of this data. Entire differential expression analysis pipelines have been built specifically for analyzing data generated in differential expression studies. These pipelines often end at the differential expression analysis step, the output of which is lists of differentially expressed entities (e.g. genes, proteins, etc.) [2]. Each file represents all the entities that were differentially expressed between two conditions (e.g. control vs. drug A). In addition to the entity names themselves, fold changes, *p*-values, and many other categories of information are commonly listed within the differential expression files [3–6]. Filtering and comparing files of differentially expressed entities is a common task that is usually done by writing custom Perl, Python, or R scripts. This task can grow cumbersome when dealing with many pairwise comparisons. A-Lister addresses that concern by allowing the user to filter and compare any number of pairwise comparisons across any number of differential expression files within a single command. A-Lister accepts most common delimited (tab, comma, colon, semicolon, and space) text files containing differential expression data and is thus compatible with most differential expression tools.

A-Lister is intended for use within bioinformatics analysis pipelines between the differential expression (DE) analysis and the visualization/pathway analysis steps. A-Lister narrows down lists of differentially expressed entities produced by differential expression analysis tools. These entities can then be further analyzed using visualization, pathway, and other bioinformatics software.

## Implementation

A-Lister is written in Python 3.7. A-Lister is freely available on GitHub at [7]. The command line interface (CLI) version can be run in Windows, Mac, and Unix operating systems. The graphical user interface (GUI) version can be used to generate and launch A-Lister commands.

### Workflow and output

An A-Lister command can be written and executed directly at a command line, or the command can be generated and executed through the GUI. All relevant input is supplied within a single A-Lister command. There are two commands available: diff-expression and name-list. The diff-expression command is used to execute A-Lister in differential expression (DE) mode. The name-list command is used to execute A-Lister in generic name list mode.

**Fig. 1:**
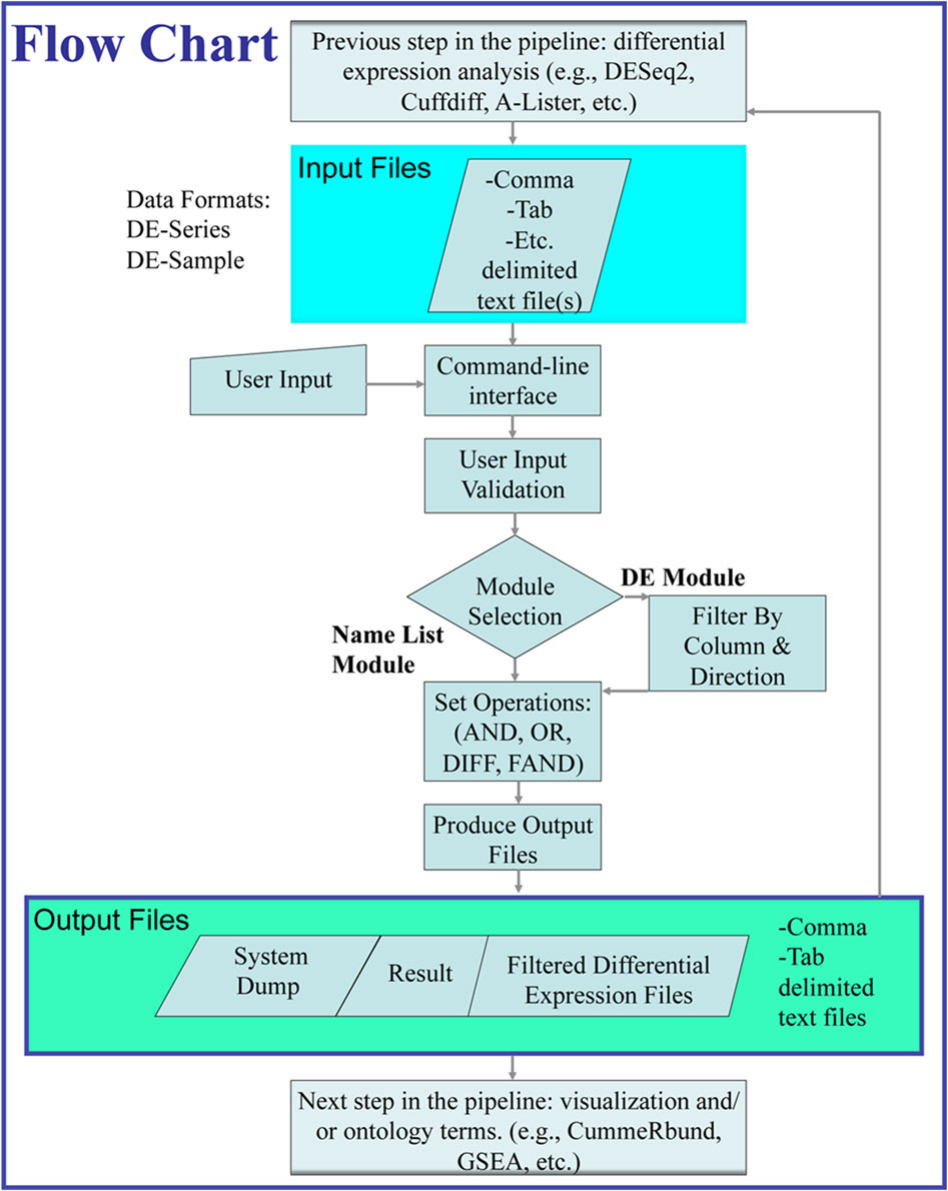
Data and control flow diagram of A-Lister

Below is a description of how A-Lister executes diff-expression and name-list commands (Fig. 1). Once the command is entered, A-Lister proceeds to validate it. If the command is valid, the program reads in the input files provided by the user. If specified by the user, the data within the input files may be filtered by any column. Furthermore, in DE mode the individual pairwise comparisons can be filtered by direction (sign of fold change). Set operations are then performed on the groups (name list mode) or pairwise comparisons (DE mode) as specified within the query. Once the query is executed, a delimited list of the resultant entity names and the count, is written into the result file. A system dump file is also output containing additional information regarding A-Lister’s execution that can be helpful with debugging or validation. Additionally, in DE mode, the filtered copies of the original input files are output. These files are obtained by filtering the original input files by the result.

### Input files

#### Input files for the name list command

In this mode, A-Lister accepts files containing columns of names delimited by tabs or commas. The header row must contain the group name for each column. An example of a name list file with three groups: control, treated1, and treated2 is shown below (Table 1). A further example of this format is supplied as an additional file (see Additional file 1: Sample input file S1).

**Table 1:** Example of a Name List File

Control	Treated1	Treated2
AADACL2	AADACP1	AADACP1
AADACL4	DUSP5P1	AMICA1

#### Input files for the differential expression command

In this mode, A-Lister accepts differential expression files containing a primary ID column (e.g. gene name), fold change column(s), and any other columns present. The columns in these files must be delimited by tab, comma, colon, semicolon, or space. A-Lister supports two types of differential expression file formats described below.

Differential Expression Sample Format (DE-Sample) (Row-Format) File: This is a delimited text file containing a primary ID column, single Fold Change column, one Sample1 column, and one Sample2 column. The Sample1 and Sample2 columns identify to which pairwise comparison each row belongs. In this way, multiple pairwise comparisons can be listed within a single DE-Sample file using a single fold change column (Table 2). The .diff files that are output from Cuffdiff follow this format [8] (see Additional file 2: Sample input file S2).

**Table 2:** Example of a DE-Sample File

gene	locus	sample1	sample2	log2(FC)	*p*-value
FAM3A	chrX:154506158–154,516,242	q1	q2	2.73	0.0023
FAM3A	chrX:154506158–154,516,242	q3	q4	0.0649976	0.81

Differential Expression Series Format (DE-Series) (Column-Format) File: This is a delimited text file containing a single ID column and multiple Fold Change columns. Each Fold Change column contains data pertaining to a single pairwise comparison. In this way, multiple pairwise comparisons can be listed within a single file using multiple fold change columns (Table 3). This is the most common format for differential expression files (see Additional file 3: Sample input file S3).

**Table 3:** Example of a DE-Series File

gene	locus	log2(FC)	*p*-value	log2(FC)2	*p*-value2
FAM3A	chrX:154506158–154,516,242	2.73	0.0023	0.0649976	0.81

### A-Lister filtering

A-Lister filtering is performed if the user specifies the optional filter by column (−f) parameter (Table 4) for any column (attribute) within a differential expression file. When filtering a DE-Sample file by an attribute the entire file is filtered. When filtering a DE-Series file by an attribute, there are two possible behaviors. First, if the filter attribute belongs to a pairwise comparison, such as p-value2, then only that pairwise comparison is filtered. Second, if the filter attribute belongs to the entire file (e.g. ID column), then the entire file is filtered. Additionally, pairwise comparisons can be filtered by direction (sign of fold change) using the directional query (−dq) argument described below (Table 4).

**Table 4:** A-Lister Command Line Interface (CLI)

Command	Argument	Brief Description
name-list	**<input-file>**	Full path to the input file.
	**<query>**	The query to be performed over input name lists.
	-id	Delimiter used in input file.
	-o	Output directory.
	-od	Delimiter used in output file.
	-v	Verbose flag.
	-e, −-examples	Show examples and exit.
	-h, −-help	Show help/manual and exit.
diff-expression	**<input-file>**	Full path to the input file.
	**-dq, <direct-query>**	The directional query to be performed over pairwise comparisons.
	**-pc, <pc-mapping>**	Specifies the layout of pairwise comparison within the file.
	-n	Specifies the ID column in file.
	-fc	Specifies the fold change column(s) in file.
	-s1	Specifies sample1 column in file.
	-s2	Specifies sample2 column in file.
	-f	Filter parameter used to filter the files by columns/attributes.
	-id	Delimiter used in input file.
	-o	Output directory.
	-od	Delimiter used in output file.
	-v	Verbose flag.
	-e, −-examples	Show examples and exit.
	-h, −-help	Show help/manual and exit.

Bolded parameters are mandatory

### A-Lister directional query

A-Lister directional query is composed of pairwise comparisons, set operators, and optional directions. The pairwise comparison names are derived from the pairwise comparison mapping argument (−pc) (Table 4). The permitted set operators are: AND, FAND, OR, and DIFF. Additionally, parenthesis can be used to nest and to set order of operations. A directional query is specified with the (−dq) argument used in the diff-expression command (Table 4).

### Set operations

Specifying the AND operator on two sets of elements returns a set of all the elements that are present within both sets. The FAND operator applied to two sets returns a set of all the similar elements from within both sets. A customized Jaro-Winkler algorithm is used to calculate similarity. To be considered similar, two strings must have Jaro-Winkler score > 0.84 [9]. The OR operator applied to two sets returns all the elements present in either set. The DIFF operator applied to two sets returns all the elements present in the first set, but not in the second. All set operations are implemented using the standard Python library.

### Directionality

Specifying the UP keyword in a query selects all entities whose fold change values are positive for a given pairwise comparison. Specifying DOWN in a query selects all entities whose fold change values are negative for a given pairwise comparison. ALL is a special modifier that results in multiple queries. That is, query results are returned as if ALL was specified as all combinations of UP and DOWN. For example, a query containing N ALL directions is transformed into 2^N^ queries. Each query is then executed and the results for each query are output into the output files in separate directories. NONE is the default direction for all pairwise comparisons. Pairwise comparisons with NONE direction are not filtered by direction.

#### A-Lister query (non-directional)

A non-directional query is composed of group names and set operators. The set operators are the same as in the directional query (e.g., AND, FAND, OR, DIFF), and can also include parentheses to nest and order the operators. The group names are derived from the first (header) row of the name list files. The non-directional query argument is used in the name-list command (Table 4).

## Results

A-Lister can be executed through a command line interface (CLI) or a graphical user interface (GUI). Underlying A-Lister’s CLI and GUI is organization into two commands. The two commands are name-list and diff-expression, which represent the generic name list mode and the differential expression mode of execution. Each command has its own set of arguments (Table 4) (see Additional file 4: User Manual). We will first describe the CLI through example use cases to illustrate the parameters and functionality, and then given an overview of the GUI version.

### Use case 1: analysis of name list files and fuzzy intersection (FAND) operation

Suppose the user wants to identify all same and similar genes within two sets of genes (Table 5). The first set is contained in file A and the second set is contained in file B (see Additional file 5: Name-List file A, Additional file 6: Name-List file B).

**Table 5:** Example of Name-List Command with Intersection (AND) and Fuzzy Intersection (FAND) Query

Set1	Set2	AND (same)	FAND (similar)
ACTA2	ACTA2	ACTA2	ACTA2
ACTG1	ACTN1	ACTN1	ACTG1
ACTN1	ADAMTS1	ADAMTS1	ACTN1
ACAD9	ERVV-2	FLNB	ADAMTS1
ADAMTS1	FLNB	KLF4	ADAMTS9
ADAMTS9	KDM4A	PIK3R3	FLNA
EXD1	KLF4	SLC20A1	FLNB
FLNA	KLF6	SLC25A25	KLF10
FLNB	MYOM1		KLF2
KLF10	PIK3R3		KLF4
KLF2	SLC19A2		KLF6
KLF4	SLC20A1		PIK3IP1
OR1S1	SLC25A25		PIK3R3
PIK3IP1	TNFRSF12A		SLC12A2
PIK3R3	ZIM2		SLC19A2
SLC12A2			SLC20A1
SLC20A1			SLC25A25
SLC25A25			TNFRSF12A
TNFSF10			TNFSF10
ZFAT-AS1			

The A-Lister command listed below will provide the same genes within the 2 files by using the AND operator:

*python ALister_CLI.py name-list “Set1-AND-Set2” FileA.txt FileB.txt -o E:/Data/Sample_Output*

The A-Lister command listed below will provide the similar genes within the 2 files by using the FAND operator:

*python ALister_CLI.py name-list “Set1-FAND-Set2” FileA.txt FileB.txt -o E:/Data/Sample_Output*

The output of these commands is shown in Table 5.

### Use case 2: analysis of differential expression using a complex query

**Fig. 2:**
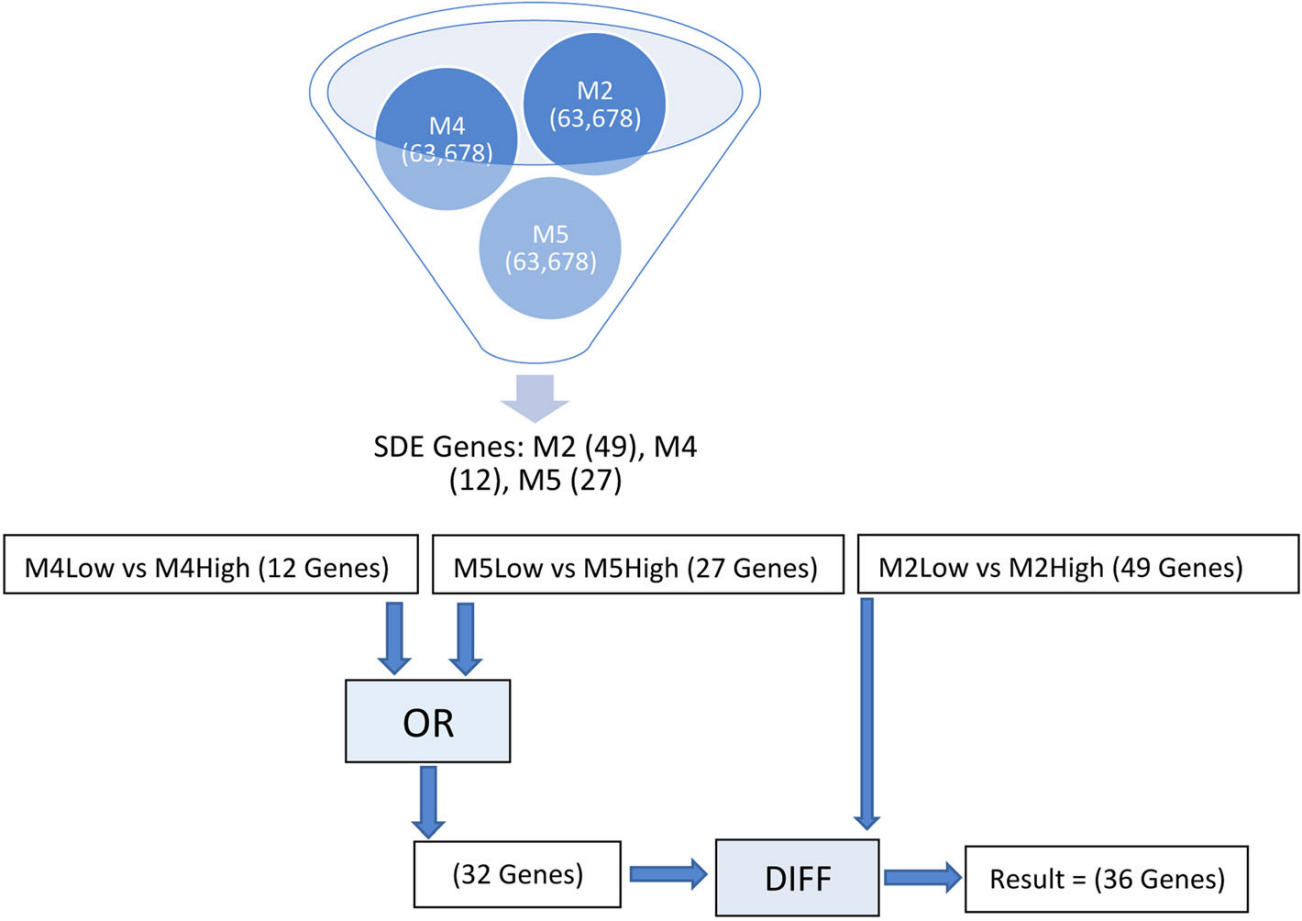
Data and process flow chart for use case 2. Input Files M2, M4, and M5 each contain 65,678 rows of differentially expressed genes. A-Lister is used to filter these files by abs(log2(foldchange)) > 1.0. The filtered files are then processed by A-Lister with the OR and DIFF set operators, resulting in 36 genes

The data for this use case can be downloaded from NCBI’s gene expression omnibus (GEO) database [10]. The series number is GSE126785 [11]. There are three groups of samples in the study: two types of induced pluripotent stem cells (iPSCs) and embryonic stem cells (ESCs). The gene expression of each group was measured under 5% oxygen and under 20% oxygen. The published files are three DESeq2 files, each containing genes differentially expressed for a single cell line between the 5% oxygen and 20% oxygen conditions. M2 is the ESC line. M4 and M5 are the iPSC lines. Suppose the user wants to know which genes are significantly differentially expressed in the embryonic stem cells (under different oxygen conditions) but are not significantly differentially expressed in either of the induced pluripotent stem cells (Fig. 2).

The A-Lister command listed below will provide the resulting genes:

python ALister_CLI.py diff-expression GSE126785_M2.txt GSE126785_M4.txt GSE126785_M5.txt -pc “M2Low*M2High- > 3.log2(FC)” “M4Low*M4High- > 3.log2(FC)” “M5Low*M5High- > 3.log2(FC)” -dq “M2Low*M2High-DIFF-(M5Low*M5High-OR-M4Low*M4High)” -o E:/Data/Sample_Output/ -n “1.GeneID” -f “3.log2(FC):agt1.0” -fc “3.log2(FC)” -v

There are three input files. Each file contains a single pairwise comparison that is mapped to its corresponding fold change column within the -pc argument. An output directory is specified using the optional -o argument. The ID and fold change columns are identified for each file using the -n and -fc flags. Each file is filtered according to the fold change values, which must be greater than 1 or less than − 1. The A-Lister directional query is specified within the -dq argument. The result file (see Additional file 7: Use case 2 result) containing thirty-six genes that satisfied the query and passed the filters can be found in the additional files.

### Use case 3: analysis of differential expression using directionality patterns

The data for this use case can be downloaded from National Center for Biotechnology Information’s (NCBI’s) gene expression omnibus (GEO) database [10]. The series number is GSE108643 [12]. There are two groups of participants in the study: lean individuals and overweight/obese individuals. Muscle biopsies were collected from both groups before and after exercise. RNA-seq data was generated on the Illumina platform, TopHat was used for sequence alignment, and Cuffdiff was used for differential gene expression analysis.

The Cuffdiff files contain four conditions: LeanPre, LeanPost, OvobPre, OvobPost. Each condition is compared to every other condition resulting in six pairwise comparisons: LeanPre vs. LeanPost, LeanPre vs. OvobPre, LeanPre vs. OvobPost, LeanPost vs. OvobPre, LeanPost vs. OvobPost, and OvobPre vs. OvobPost. Suppose the user wants to examine which genes are significantly upregulated in both lean and overweight/obese individuals post exercise. The A-Lister command listed below will provide the resulting genes:

python ALister_CLI.py diff-expression GSE108643_Cuffdiff.txt -pc “LeanPre- > LPE,LeanPost- > LPO,OvobPre- > OPE,OvobPost- > OPO” -dq “LPE*LPO:UP-AND-OPE*OPO:UP” -f “log2(fold_change):agt1.0,q_value:lt0.05,value_1:gt1.0,value_2:gt1.0” -s1 “sample_1” -s2 “sample_2” -n “gene”

A diff-expression command will be executed with the *GSE108643_Cuffdiff.txt* input file. Each file specific condition label is mapped to a globally unique label within the -pc argument. This mapping is important when dealing with multiple files that contain the same condition label names (e.g. q1, q2, q3, etc.) or, as in this example, when the user would like to shorten the name to avoid typing long group names. The -s1, −s2, and -n arguments specify the names of sample1, sample2, and ID columns. In this example, the -f argument will be used to filter the file according to absolute value of log2(fold change) greater than 1.0 (agt1.0), q-value less than 0.05 (lt0.05), values 1 and 2 greater than 1.0 (gt1.0). The A-Lister query is specified within the -dq argument, where LPE*LPO represents lean pre-exercise vs. lean post-exercise, and OPE*OPO represents overweight/obese pre exercise vs. overweight/obese post exercise. Since no output directory was specified, the result is output in the result.txt file within the current working directory.

Now, suppose the user wants to examine all possible directionality patterns for the above-mentioned query. The four possible patterns are up, up; up, down; down, up; and down, down. This could be accomplished by changing the directions within the -dq argument from UP to ALL. This would result in the following A-Lister command:

python ALister_CLI.py diff-expression GSE108643_Cuffdiff.txt -pc “LeanPre- > LPE,LeanPost- > LPO,OvobPre- > OPE,OvobPost- > OP2O” -dq “LPE*LPO:ALL-AND-OPE*OPO:ALL” -f “log2(fold_change):agt1.0,q_value:lt0.05,value_1:gt1.0,value_2:gt1.0” -s1 “sample_1” -s2 “sample_2” -n “gene”

This is an ALL query (a query containing an ALL directionality) with two ALL directions, so it is effectively translated into four queries: LPE*LPO:UP-AND-OPE*OPO:UP, LPE*LPO:UP-AND-OPE*OPO:DOWN, LPE*LPO:DOWN-AND-OPE*OPO:UP, LPE*LPO:DOWN-AND-OPE*OPO:DOWN. Since no output directory was specified, the results for all four queries are output in the result.txt file within the current working directory.

**Fig. 3:**
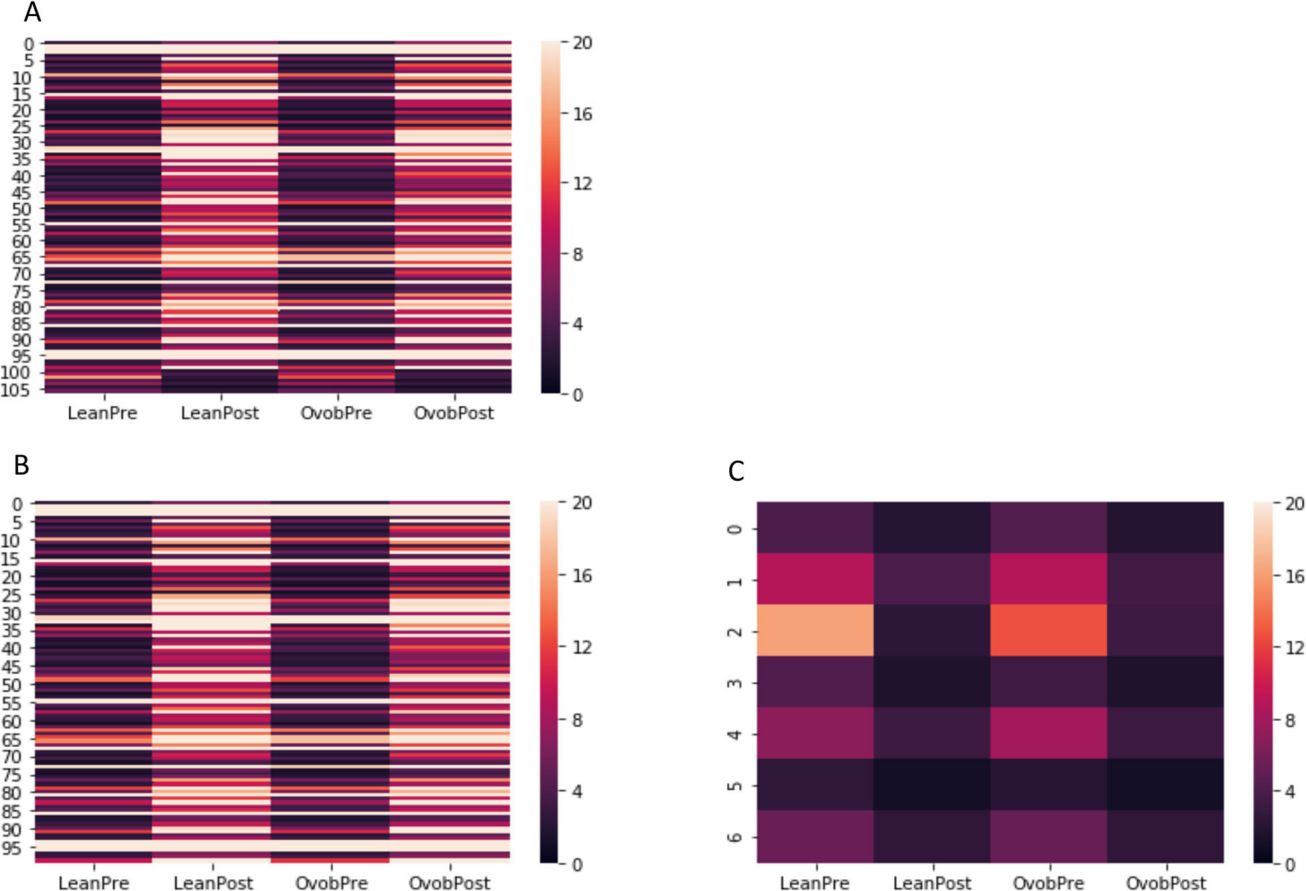
Heatmap visualization of significantly differentially expressed genes for use case 3. **a** All genes that are significantly differentially expressed for both LeanPre vs. LeanPost and OvobPre vs. OvobPost pairwise comparisons. **b** All genes that are significantly upregulated in both pairwise comparisons. **c** All genes that are significantly downregulated in both pairwise comparisons

This example found one hundred seven genes are differentially expressed in both LPE*LPO and OPE*OPO pairwise comparisons (Fig. 3). One hundred genes are upregulated and seven genes are downregulated in both of these pairwise comparisons. Zero genes are upregulated within one of these pairwise comparisons while also being upregulated in another one of these pairwise comparisons. The result file (see Additional file 8: Use case 3 result) containing the upregulated and downregulated genes can be found in additional files.

### Graphical user Interface (GUI)

**Fig. 4:**
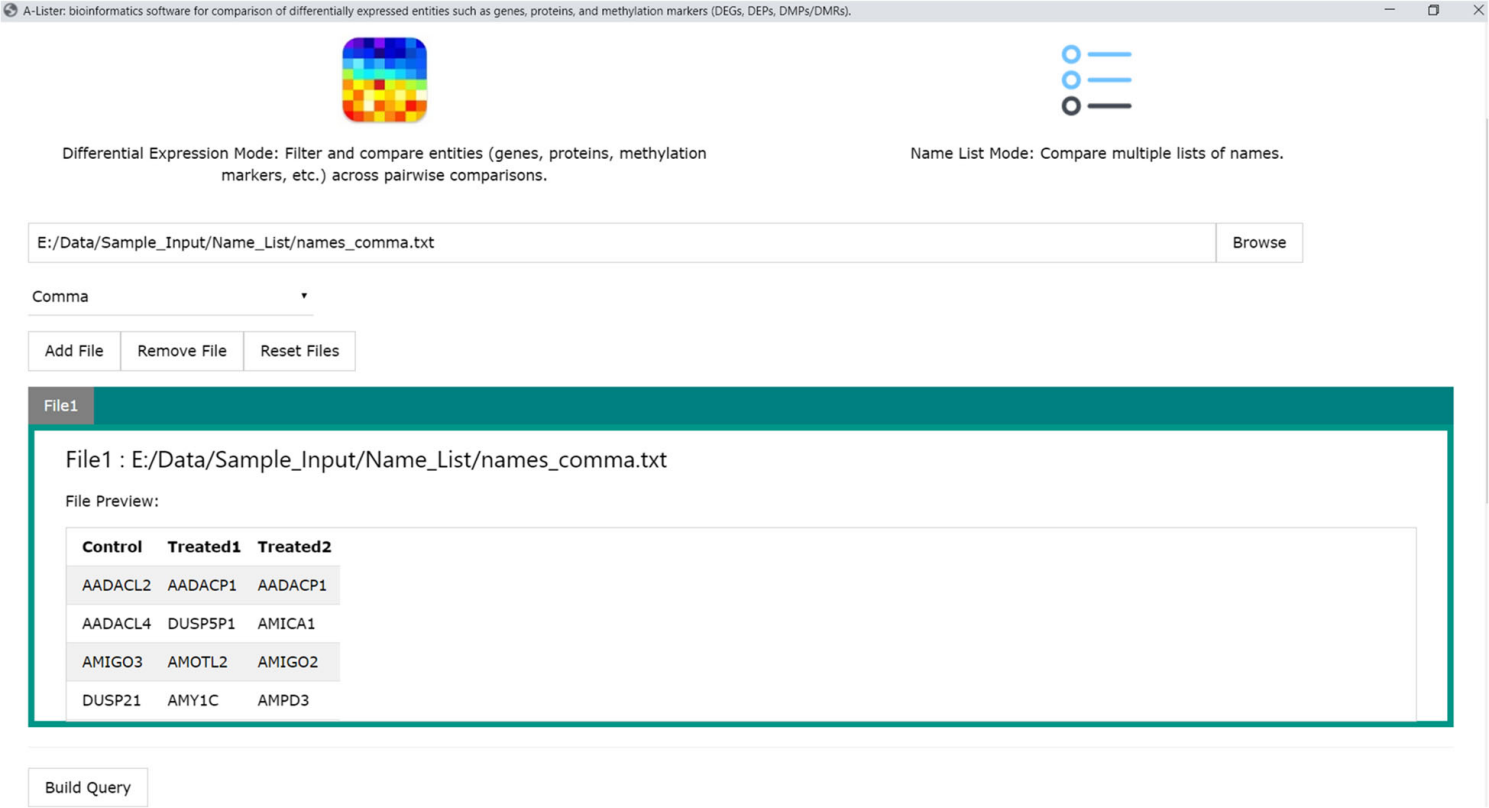
Example of screenshot of Graphical User Interface (GUI) version of A-Lister

The GUI guides the user through creating a command necessary to run A-Lister with desired settings. After selecting the mode (differential expression or name list), the parameters for that mode will be presented. The user will browse for files and preview the column headings and the first few lines of each input file in order to facilitate setting the filtering and mapping parameters. If appropriate for the mode, the GUI will also enable selecting the comparison groups, directionality, and set operators, necessary for creating the query. Once the parameters for all files are set, the user can generate and launch the command. Detailed instructions on the use of the GUI can be found in the ReadMe.pdf file (Additional file 4), and an example screenshot of the GUI is shown (Fig. 4).

## Discussion

Although several existing bioinformatics tools have some overlapping functionality with A-Lister, none fill the same role as A-Lister. Several such tools are listed in Table 6 and are described below. Intervene is a tool that can compute and visualize intersections of gene sets (or genomic regions) using multiple visualization techniques such as Venn diagrams, UpSet plots, and heatmaps [15]. VennPainter and InteractiVenn are similar to Intervene [16, 17]. Statistical R packages SuperExactTest and Gene-Overlap package can also be used to compute and visualize intersections of sets [18, 19]. Galaxy suite text manipulation tools can be used to filter and compare tab delimited text files [14]. The key limitation of these tools is that they are not built to deal with differential expression data specifically. As such the above-mentioned tools lack the means to filter individual pairwise comparisons.

**Table 6:** Comparison to existing software

Application	Filters	Query	Set Operators	Input Type	Interface
A-Lister	yes	yes	AND, FAND, OR, DIFF	DE data, lists	Command Line and Graphical User Interface
Functional Heatmap [13]	yes	no	AND (implicit)	DE timeseries data	Web App
Galaxy (Text Manipulation) [14]	yes	no	AND, OR, DIFF	Tabular data	Web App
Intervene [15]	no	no	AND	Genomic regions, binary, counts, lists	Command Line, Web App
VennPainter [16]	no	no	AND	Lists	Graphical User Interface
InteractiVenn [17]	no	no	AND	Lists	Web App
SuperExactTest [18]	no	no	AND, OR	Lists	R
Gene-Overlap [19]	no	no	AND, OR	Lists	R

Functional Heatmap is another novel tool that seeks to make filtering and comparison of lists of differentially expressed entities less cumbersome [13]. Similar to A-Lister, Functional Heatmap allows the user to filter differential expression data by columns (e.g. *p*-value) and by direction (sign and magnitude of fold change). However, Functional Heatmap is specialized for analyzing time-series data, specifically analyzing patterns of fold change direction across time. A-Lister, on other hand, can be used to analyze any pairwise comparison differential expression data across conditions, tissues, and timepoints. Moreover, unlike Functional Heatmap, A-Lister supports the notion of queries. The queries allow the user to quickly examine complex relationships between pairwise comparisons (Table 6).

In the future we plan to add ID validation and mapping in order to enable integration of different DEE types. Studies containing multiple -omics types are increasingly common, and we would like to be able to seamlessly compare genes, proteins, and methylation markers with each other. However, the names used as IDs for the different data types (genes, proteins, methylation markers) are generally not the same, and rather, are dependent on the data type naming convention and database. The IDs often do not map one to one, but rather one to many or one to none. Even within one data type, such as gene expression data, there are differences in naming due to annotation version, platform used, species, and other characteristics. Currently, UniProt provides a web-based tool (Retrieve/ID mapping tool) to convert IDs between different annotations [20]. To maintain A-Lister’s lightweight requirements (e.g., the user does not have to download datasets), and offline capabilities for secure human data processing, we did not implement linking with databases through local or outside connections to web services to check or query IDs. We do recommend that users interested in such capability should initially process the data names in their files through a service such as UniProt before executing A-Lister. In the future, we may implement and host a web server to facilitate this functionality and to make A-Lister more accessible. However, these features will require the addition of complex name mapping functionality and web back-end to A-Lister, which we propose as a future enhancement.

## Conclusions

A-Lister allows the user to quickly filter and compare any number of pairwise comparisons across multiple heterogenous differential expression files. Additionally, the A-Lister can be used to examine patterns of fold change direction and to execute complex queries across multiple pairwise comparisons. This tool may be especially useful in the context of data mining applications where dealing with many heterogenous files is common. A-Lister will help researchers to save time spent on writing, maintaining, and adjusting custom differential expression analysis scripts.

## Availability and requirements

**Project name:** A-Lister.

**Project home page:** https://github.com/staslist/A-Lister

**Operating system(s):** Windows, Mac OS (10.10.x+), Unix.

**Programming language:** Python.

**Other requirements:** Python 3.7+, Google Chrome 76.0+, Eel 0.10.4.

**License:** MIT.

**Any restrictions to use by non-academics:** None.

## Supplementary information

**Additional file 1.** Name-List sample input file.

**Additional file 2.** DE-Sample sample input file.

**Additional file 3.** DE-Series sample input file.

**Additional file 4.** User Manual.

**Additional file 5.** Name-List file A.

**Additional file 6.** Name-List file B.

**Additional file 7.** Use case 2 result.

**Additional file 8.** Use case 3 result.

**Additional file 9.** A-Lister source code.

## Data Availability

The datasets analyzed in the examples shown above in the manuscript are available in the Gene Expression Omnibus repository under accession numbers GSE126785 and GSE108643, [https://www.ncbi.nlm.nih.gov/geo/] [11, 12]. The source code is available at https://github.com/staslist/A-Lister and as additional file (see Additional file 9: A-Lister source code).

## Abbreviations

CLI: Command line interface
DE: Differential expression
DEE: Differentially expressed entity
DEG: Differentially expressed gene
DEP: Differentially expressed protein
DMP: Differentially methylated position
DMR: Differentially methylated region
ESC: Embryonic stem cell
GEO: Gene expression omnibus
GUI: Graphical user interface
iPSC: Induced pluripotent stem cell
NBCI: National center for biotechnology

## References

[1] Stephens ZD, Lee SY, Faghri F, Campbell RH, Zhai CX, Efron MJ, Iyer R, Schatz MC, Sinha S, Robinson GE (2015). Big Data: Astronomical or Genomical?. Plos Biol.

[2] Conesa A, Madrigal P, Tarazona S, Gomez-Cabrero D, Cervera A, McPherson A, Szczesniak MW, Gaffney DJ, Elo LL, Zhang XG (2016). A survey of best practices for RNA-seq data analysis. Genome Biol.

[3] Efstathiou G, Antonakis AN, Pavlopoulos GA, Theodosiou T, Divanach P, Trudgian DC, Thomas B, Papanikolaou N, Aivaliotis M, Acuto O (2017). ProteoSign: an end-user online differential proteomics statistical analysis platform. Nucleic Acids Res.

[4] Love MI, Huber W, Anders S (2014). Moderated estimation of fold change and dispersion for RNA-seq data with DESeq2. Genome Biol.

[5] Robinson MD, McCarthy DJ, Smyth GK (2010). edgeR: a bioconductor package for differential expression analysis of digital gene expression data. Bioinformatics.

[6] Yassi M, Davodly ES, Shariatpanahi AM, Heidari M, Dayyani M, Heravi-Moussavi A, Moattar MH, Kerachian MA (2018). DMRFusion: a differentially methylated region detection tool based on the ranked fusion method. Genomics.

[7] Listopad S. A-Lister. https://github.com/staslist/A-Lister. Accessed 19 Sept 2019.

[8] Trapnell Cole, Hendrickson David G, Sauvageau Martin, Goff Loyal, Rinn John L, Pachter Lior (2012). Differential analysis of gene regulation at transcript resolution with RNA-seq. Nature Biotechnology.

[9] Christen P: A comparison of personal name matching: Techniques and practical issues. Icdm 2006: Sixth Ieee international conference on data mining, Workshops 2006:290–294.

[10] Edgar R, Domrachev M, Lash AE (2002). Gene expression omnibus: NCBI gene expression and hybridization array data repository. Nucleic Acids Res.

[11] Spyrou James, Gardner David K., Harvey Alexandra J. (2019). Metabolism Is a Key Regulator of Induced Pluripotent Stem Cell Reprogramming. Stem Cells International.

[12] Deyarshi PM, Jones AD, Campbell WW, Taylor EM, Henagan TM (2017). Effects of acute aerobic exercise on whole genome nucleosome maps and gene expression in skeletal muscle of lean Vs overweight/obese men. FASEB J.

[13] Williams JR, Yang RT, Clifford JL, Watson D, Campbell R, Getnet D, Kumar R, Hammamieh R, Jett M (2019). Functional Heatmap: an automated and interactive pattern recognition tool to integrate time with multi-omics assays. Bmc Bioinformatics.

[14] Afgan E, Baker D, Batut B, van den Beek M, Bouvier D, Cech M, Chilton J, Clements D, Coraor N, Gruning BA (2018). The galaxy platform for accessible, reproducible and collaborative biomedical analyses: 2018 update. Nucleic Acids Res.

[15] Khan A, Mathelier A (2017). Intervene: a tool for intersection and visualization of multiple gene or genomic region sets. Bmc Bioinformatics.

[16] Lin GL, Chai J, Yuan S, Mai C, Cai L, Murphy RW, Zhou W, Luo J (2016). VennPainter: A Tool for the Comparison and Identification of Candidate Genes Based on Venn Diagrams. PLoS One.

[17] Heberle H, Meirelles GV, da Silva FR, Telles GP, Minghim R. InteractiVenn: a web-based tool for the analysis of sets through Venn diagrams. Bmc Bioinformatics. 2015;16(1):169. 10.1186/s12859-015-0611-3 PMC4455604 25994840

[18] Wang MH, Zhao YZ, Zhang B (2015). Efficient test and visualization of multi-set intersections. Sci Rep-Uk.

[19] Shen L: GeneOverlap: An R package to test and visualize gene overlaps. https://bioconductor.org/packages/release/bioc/html/GeneOverlap.html. Accessed 19 Sept 2019.

[20] Bateman A, Martin MJ, Orchard S, Magrane M, Alpi E, Bely B, Bingley M, Britto R, Bursteinas B, Busiello G (2019). UniProt: a worldwide hub of protein knowledge. Nucleic Acids Res.

