# Supplementary material for: A-Lister: a tool for analysis of differentially expressed omics entities across multiple pairwise comparisons
Source: BMC Bioinformatics. 2019 Nov 19;20:595. doi: 10.1186/s12859-019-3121-x (PMC6862834; doi:10.1186/s12859-019-3121-x)
Supplement: Supplementary file 4 — Additional file 4. User Manual. [file 12859_2019_3121_MOESM4_ESM.pdf]

ReadMe.md

A-Lister v1.1

A-Lister is a dual interface (CLI & GUI) tool that assists with analysis of differentially expressed entities (DEEs), such as differentially expressed genes (DEGs), differentially expressed proteins (DEPs), and differentially methylated positions/regions (DMPs/DMRs), across multiple pairwise comparisons.

Installation

Supported Operating Systems: Windows 10, Mac OS (10.10.x+), Unix (Ubuntu, Other).

1. Install Python version 3.7 or higher. https://www.python.org/downloads/
2. Check whether you have pip installed. Type pip (or pip3) -V in command line (Windows), terminal (Mac OS), bash shell (Unix). If you do not have pip installed follow these instructions to install pip (python package management system). https://pip.pypa.io/en/stable/installing/
3. Install Eel. https://github.com/ChrisKnott/Eel

```
pip install eel
or
pip3 install eel
```

4. Install Google Chrome.
5. Download A-Lister from GitHub.

Running A-Lister

To run A-Lister using command line interface (CLI) type `python ALister_CLI.py` while located in A-Lister's directory. This will bring up the help menu with list of available commands. For more information please refer to command line interface documentation below.

To run A-Lister using graphical user interface (GUI) type `python ALister_GUI.py` while located in A-Lister's directory. For more information please refer to graphical user interface documentation below.

We recommend that new users use the GUI to become familiar with A-Lister. The GUI portion of documentation will guide you through complete examples. The CLI is available for those that prefer using command line.

Note: Depending on how python is setup on your system you may need to type python3 instead of python within the terminal.

About/Documentation

1. A-Lister Input

Supported File Formats: Theoretically any text delimited file should work. However, A-Lister has only been tested with standard .csv, .tsv, .txt (delimited), and .diff files.

Name List Mode:

In this mode A-Lister accepts files containg columns of names delimited by tab or comma. The header row musts contain group names for each column. Examples of this format can be found in Sample_Input/Name_List folder.

Control	Treated1	Treated2

Control	Treated1	Treated2
AADACL2	AADACP1	AADACP1
AADACL4	DUSP5P1	AMICA1

Differential Expression Mode:

In this mode, A-Lister accepts differential expression files containing a primary id column (e.g. gene name), fold change column(s), and any other columns present. The columns in these files must be delimited by tab, comma, colon, semicolon, or space. A-Lister supports two types of differential expression file formats described below.

1. *Differential Expression Sample Format (DE-Sample) (Row-Format) File*:
   This is a delimited text file containing a primary ID column, single Fold Change column, one Sample1 column, and one Sample2 column. The Sample1 and Sample2 columns identify to which pairwise comparison each row belongs. This way multiple pairwise comparisons can be listed within the same DE-Sample file using a *single* fold change column. Examples of this format can be found in Sample_Input/DE_Sample/ folder.

gene	locus	sample_1	sample_2	log2(FC)	p_value
FAM3A	chrX:154506158-154516242	q1	q2	2.73	0.0023
FAM3A	chrX:154506158-154516242	q3	q4	0.0649976	0.81

2. *Differential Expression Series Format (DE-Series) (Column-Format) File*: This is a delimited text file containing a primary ID and multiple Fold Change columns. Each Fold Change column contains data pertaining to a single pairwise comparison. This way multiple pairwise comparisons can be listed within the same DE-Series file using *multiple* fold change columns. Examples of this format can be found in Sample_Input/DE_Series/ folder.

gene	locus	log2(FC)	p_value	log2(FC)2	p_value2
FAM3A	chrX:154506158-154516242	2.73	0.0023	0.0649976	0.81

2. A-Lister Functionality

A-Lister has two primary modes of operation: name list and differential expression. In differential expression mode A-Lister can be used to compare and filter lists of differentially expressed entities (DEEs) such as DEGs, DEPs, and DMPs/DMRs. The input text delimited files containing differential expression data can be filtered by any column. A common example is filtering input data based on p-value column to only include rows with p-value <= 0.05. A-Lister assumes that the DEEs are listed in form of paired comparisons within the input files. That is every DEE in a given file belongs to some paired comparison between two conditions (e.g., treated vs control). These paired comparisons can be filtered based on the directionality (sign) of their respective fold changes. That is, all DEEs belonging to a particular paired comparison can be filtered based on the sign of their fold change.

In addition to column and directionality based filtering, A-Lister can compare paired comparisons with one another using set operations such as AND (intersect), FAND (fuzzy intersect), OR (union), and DIFF (difference). The combination of these filtering and set operations allows us to ask interesting questions (queries) about our data.

For example, suppose we have two paired comparisons in a single differential expression file or across two differential expression files. Paired comparison number one has a list of differentially expressed genes (DEGs) between the control group and group treated with drug #1. Paired comparison number two has a list of DEGs between control group and group treated with drug #2. We would like to know which genes were more highly expressed in control group than in groups treated with drug #1 and drug #2. This question can be represented by the following A-Lister query (CNTRL*T1:DOWN-AND-CNTRL*T2:DOWN). More complex queries can be constructed by comparing any number of paired comparisons from across an unlimited number of files. Order of operations can be enforced within the query by using parenthesis.

In name-list mode you can also perform set operations on lists of names. However, you cannot use any filtering operations.

A-Lister Filtering

A-Lister supports filtering differential expression files by columns (attributes). When filtering a DE-Sample file by an attribute the entire file is filtered. When filtering a DE-Series file by an attribute there are two possible behaviors. First, if the filter attribute belongs to a pairwise comparison, such as p-value2, then only that pairwise comparison is filtered. Second, if the filter attribute belongs to the entire file (e.g. ID column), then the entire file is filtered. Additionally, pairwise comparisons can be filtered by direction (sign of fold change) using the directional query argument.

A-Lister Directional Query

A-Lister directional query is composed of pairwise comparison names, set operators, and optional directions. The pairwise comparison names are derived from the pairwise comparison mapping. The permitted set operators are: AND, FAND, OR, and DIFF.

Set Operations

AND - intersection. An intersection of two sets returns a set of all the elements that are present within both sets.

FAND - fuzzy intersection. A fuzzy intersection of two sets returns a set of all the similar elements from within both sets. A customized Jaro-Winkler algorithm is used to calculate similarity. To be considered similar two strings must have have Jaro-Winkler score > 0.84.

Jaro-winkler algorithm: http://users.cecs.anu.edu.au/~Peter.Christen/publications/tr-cs-06-02.pdf

Example: [FRAC10, ALOC, BRAC] FAND [FRAC11, ALUK, PRAT14, MRA, BRAC] = [FRAC10, FRAC11, BRAC]

OR - union. A union of two sets returns all the elements present in either set.

DIFF - difference. A difference of two sets returns all the elements present in the first set, but not in the second.

Directionality

UP - upregulated. Selects all entities whose fold change values are positive for a given pairwise comparison.

DOWN - downregulated. Selects all entities whose fold change values are negative for a given pairwise comparison.

ALL is a special modifier that results in multiple queries. In one query ALL is replaced by UP. In the other query ALL is replaced by DOWN. A query containing N ALL directions is transformed into N^2 queries. Each query is then executed and the results for each query are output into the output files.

Example 1: CNTRL*T:ALL-AND-CNTRL*T2 -> CNTRL*T:UP-AND-CNTRL*T2 , CNTRL*T:DOWN-AND-CNTRL*T2.

Example 2: CNTRL*T:ALL-AND-CNTRL*T2:ALL -> CNTRL*T:UP-AND-CNTRL*T2:UP , CNTRL*T:UP-AND-CNTRL*T2:DOWN , CNTRL*T:DOWN-AND-CNTRL*T2:UP , CNTRL*T:DOWN-AND-CNTRL*T2:DOWN.

NONE - the default direction for all pairwise comparisons.

Pairwise Comparison Mapping

This is done differently depending on differential expression file type (DE-Sample or DE-Series). The goal of pairwise comparison mapping is to clearly indicate the names of pairwise comparisons and to which pairwise comparison every entry in the file belongs.

For DE-Sample files the user must map each label listed in the sample1 and sample2 columns to some unique, short, and descriptive new labels. For example, file Sample_Input/DE_Sample/GSE76453_cuffdiff_E2.diff contains two labels: q1 and q2. These labels are not descriptive and may conflict with labels contained in other differential expression files. A good mapping for these labels would be q1->Ctl, q2->ZNF143 based on GEO GSE76453 description. This mapping more clearly indicates what each condition represents. The q1*q2 pairwise comparison will then be named based on these new labels as Ctl*ZNF143.

For DE-Series files the user must come up with their own pairwise comparison names and map these names to all the columns that contain data pertaining to those pairwise comparisons. For example, file Sample_Input/DE_Series/GSE126785_M2M4.csv contains two pairwise comparisons. These pairwise comparisons can be appropriately named as M2Low*M2High and M4Low*M4High based on the GEO GSE126785 experiment description. The correct mapping would then be M2Low*M2High->log2(FC)M2,P-valueM2,P-adjM2 and M4Low*M4High->log2(FC)M4,P-valueM4,P-adjM4. One of the mapped columns must always be a fold change column. The ID columns belong to all pairwise comparisons and therefore do not need to be mapped to any individual pairwise comparison.

A-Lister Query (Non-Directional)

This is the query argument used in the name-list command. A non-directional query is composed of group names and set operators. The set operators are the same as in the directional query (i.e., AND, FAND, OR, DIFF). The group names are derived from the first (header) row of the name list files.

3. A-Lister Output

A-Lister's output is contained within several files. The result.txt file contains the name list of entities returned by the query. If an ALL direction query was requested the result file will contain a name list corresponding to each directional query generated from the all direction query. The filtered differential expression files are output in differential expression mode only. Additionally, a data_dump.txt file is output containing information about how A-Lister executed the query. This information is primarily used for validation and or debugging.

A-Lister Output Structure

For Example 2: CNTRL*T:ALL-AND-CNTRL*T2:ALL ->

Query0: CNTRL*T:UP-AND-CNTRL*T2:UP
Query1: CNTRL*T:UP-AND-CNTRL*T2:DOWN
Query2: CNTRL*T:DOWN-AND-CNTRL*T2:UP
Query3: CNTRL*T:DOWN-AND-CNTRL*T2:DOWN

Output will be:
1)result.txt
2)data_dump.txt
3)FilteredDEFiles
--a)Query0 (CNTRL*T:UP-AND-CNTRL*T2:UP)
--b)Query1 (CNTRL*T:UP-AND-CNTRL*T2:DOWN)
--c)Query2 (CNTRL*T:DOWN-AND-CNTRL*T2:UP)
--d)Query3 (CNTRL*T:DOWN-AND-CNTRL*T2:DOWN)

Graphical User Interface Documentation

This section demonstrates how to use the A-Lister graphical user interface using examples.

Name List Example

One name-list file: Sample_Input/Name_List/names_comma.txt. There are 3 groups in this file: Control, Treated1, and Treated2. We want to find entities in common between Treated1 group and a union of Control and Treated2 groups. This will result in the following A-Lister query: Treated1-AND-(Control-OR-Treated2).

1. Select the name list execution mode.
2. Select Sample_Input/Name_List/names_comma.txt file using the "Browse" button. Then select the corresponding input file delimiter (comma). Finally, click "Add File" in order to add file to the file selection area. A tab will appear. You can examine file previews (1st 5 lines of the file including column headers) within this tab to assist with setting remaining parameters.

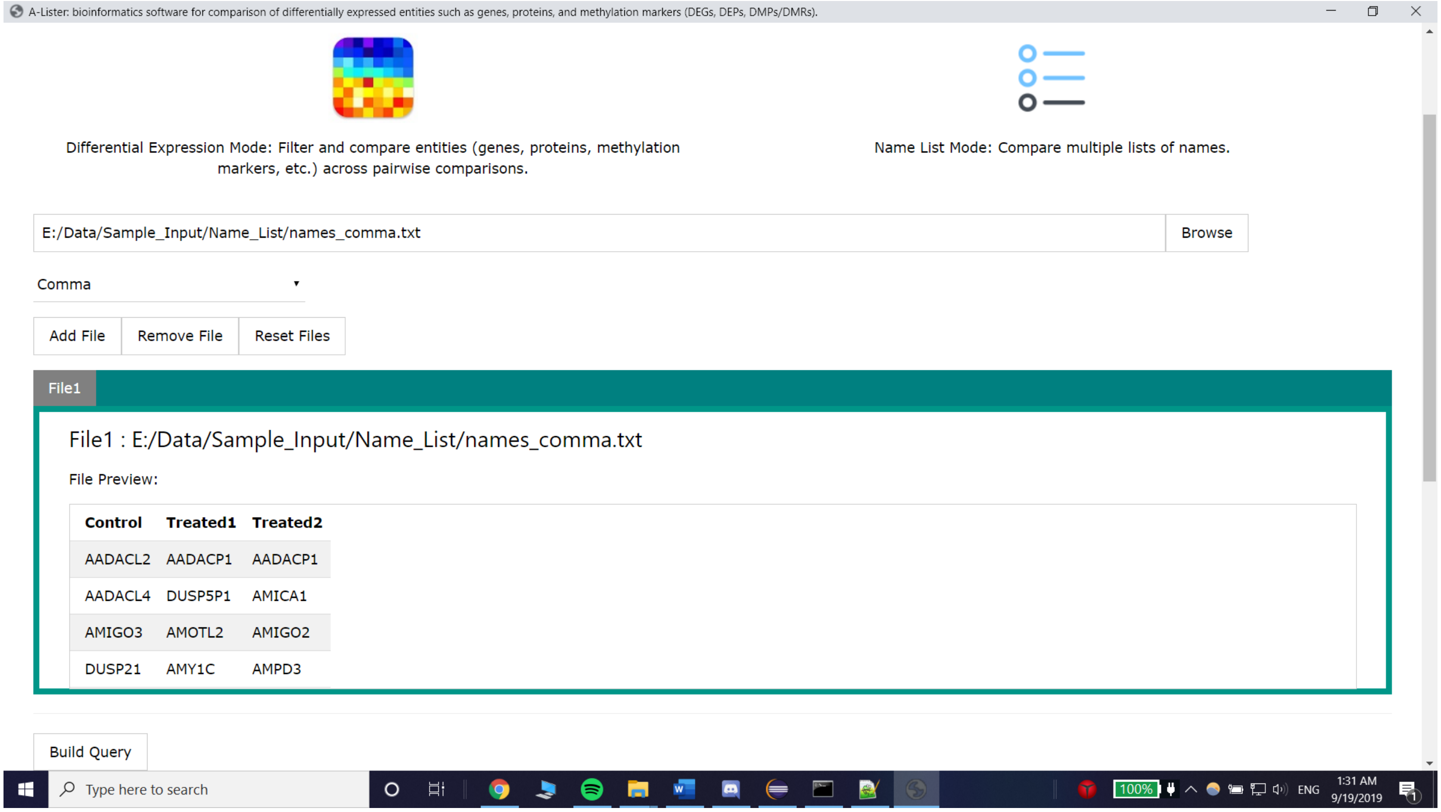

3. Either type the query into query input box or click "Build Query" and use the combination of group name and set operator buttons to build a query. Insert parenthesis into the query by typing in query box.
4. Select the output delimiter and output directory.
5. Select whether to run in verbose or silent mode. Setting verbose mode results in more information regarding A-Lister's execution being output into the data_dump.txt file.
6. Click "generate command". This generates an appropriate CLI command.

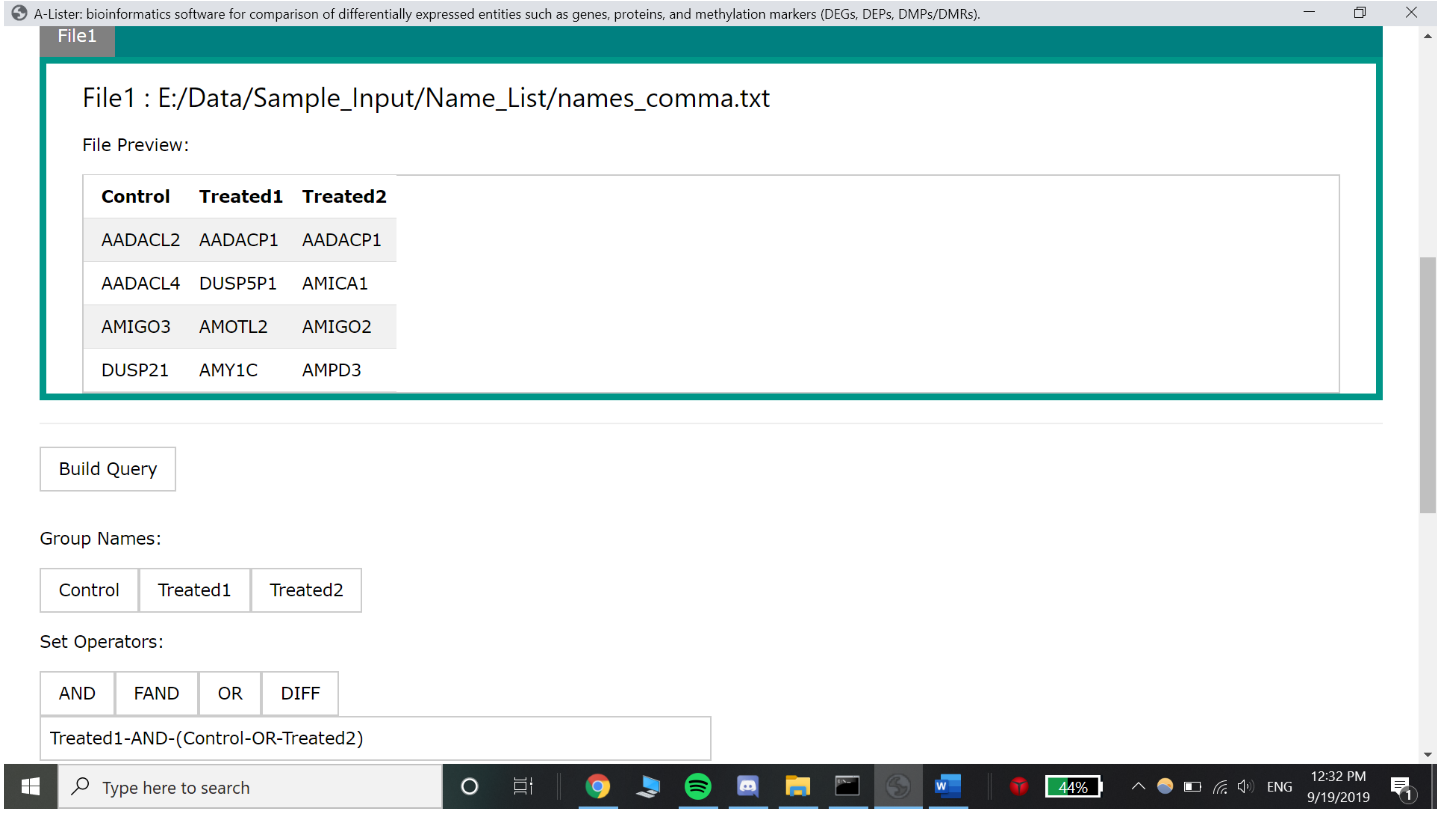

7. You can then launch this command within GUI by clicking on the "Launch" button. When executing A-Lister via the GUI a box containing the results of the query will appear at the bottom of the GUI. Alternatively, you can copy the command from the command box and paste it into the command line. The output files will be generated either way.

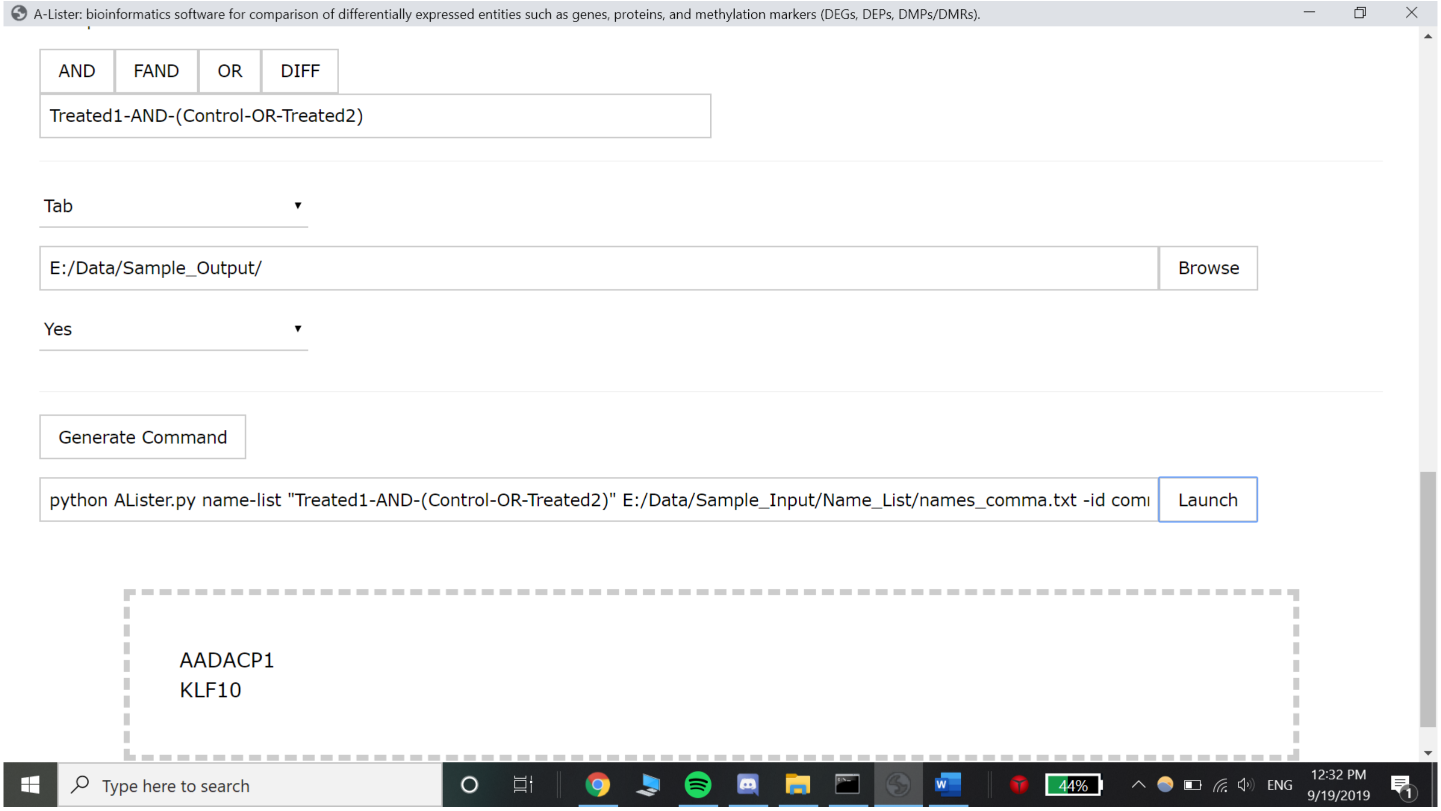

Differential Expression Example

Two differential expression files: Sample_Input/DE_Series/GSE114528_differential_exp_EVP_D.tsv and Sample_Input/DE_Sample/GSE99397_CreNeg-MHCPos_vs_CrePos-MHCNeg.diff. We want to find significantly differentially expressed genes in common between the EVP*D and CreNeg*CrePos pairwise comparisons. This will result in the following A-Lister query: EVP*D-AND-CNEG*CPOS.

1. Select the differential expression execution mode.
2. Select Sample_Input/DE_Series/GSE114528_differential_exp_EVP_D.tsv file using the "browse" button. Then select the corresponding input file delimiter (tab). Click "Add File" in order to add file to the file selection area. Click on "File1" in the file selection tab bar.

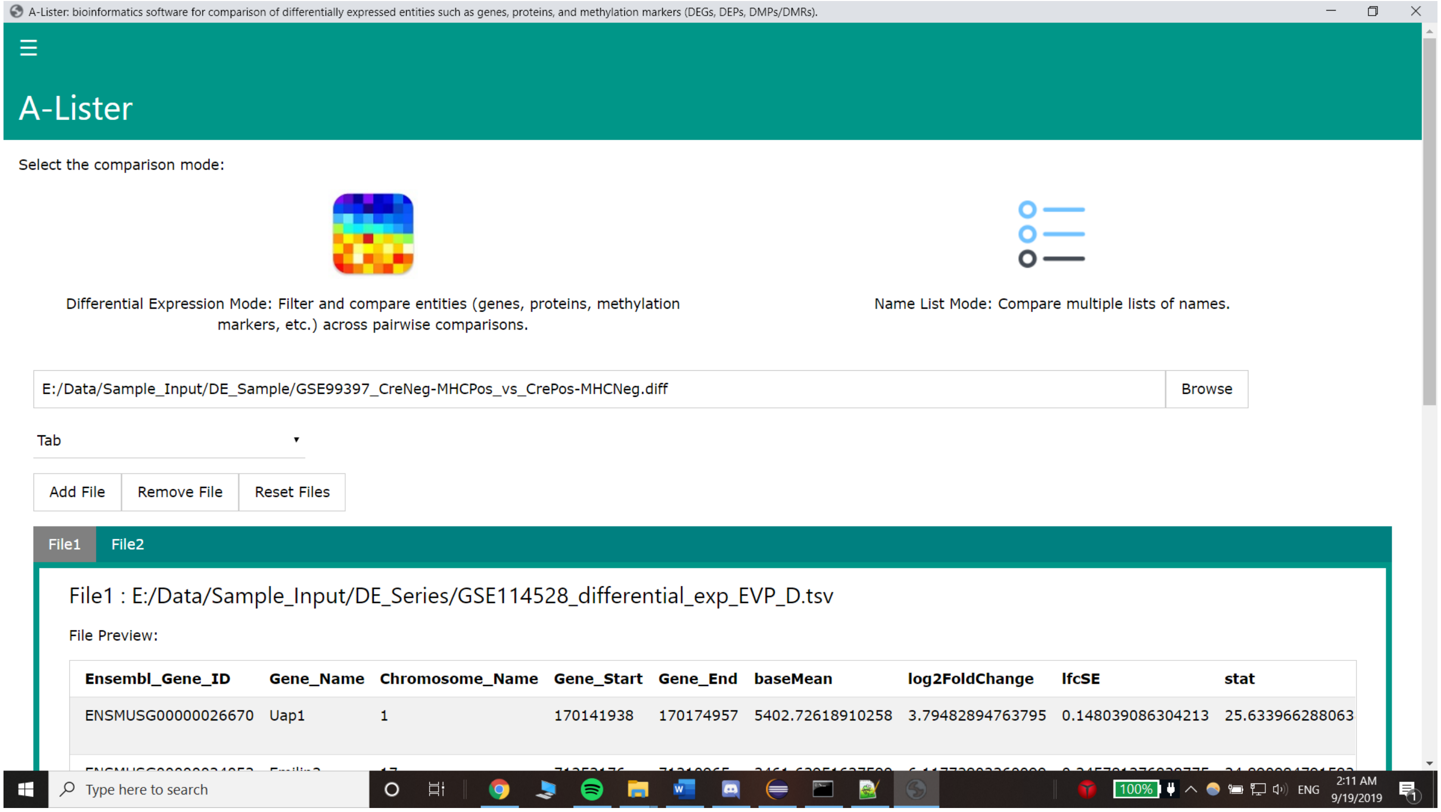

3. Select the differential expression file type (DE-Series). Verify that the ID Column Name is correct. In this example we want to use Gene_Name column. Click on "Add PC Map" in order to add a pairwise comparison mapping for this file. Name the pairwise comparison EVP*D. Then select the appropriate fold change column. If this file contains only a single pairwise comparison than it is not necessary to select additional columns.

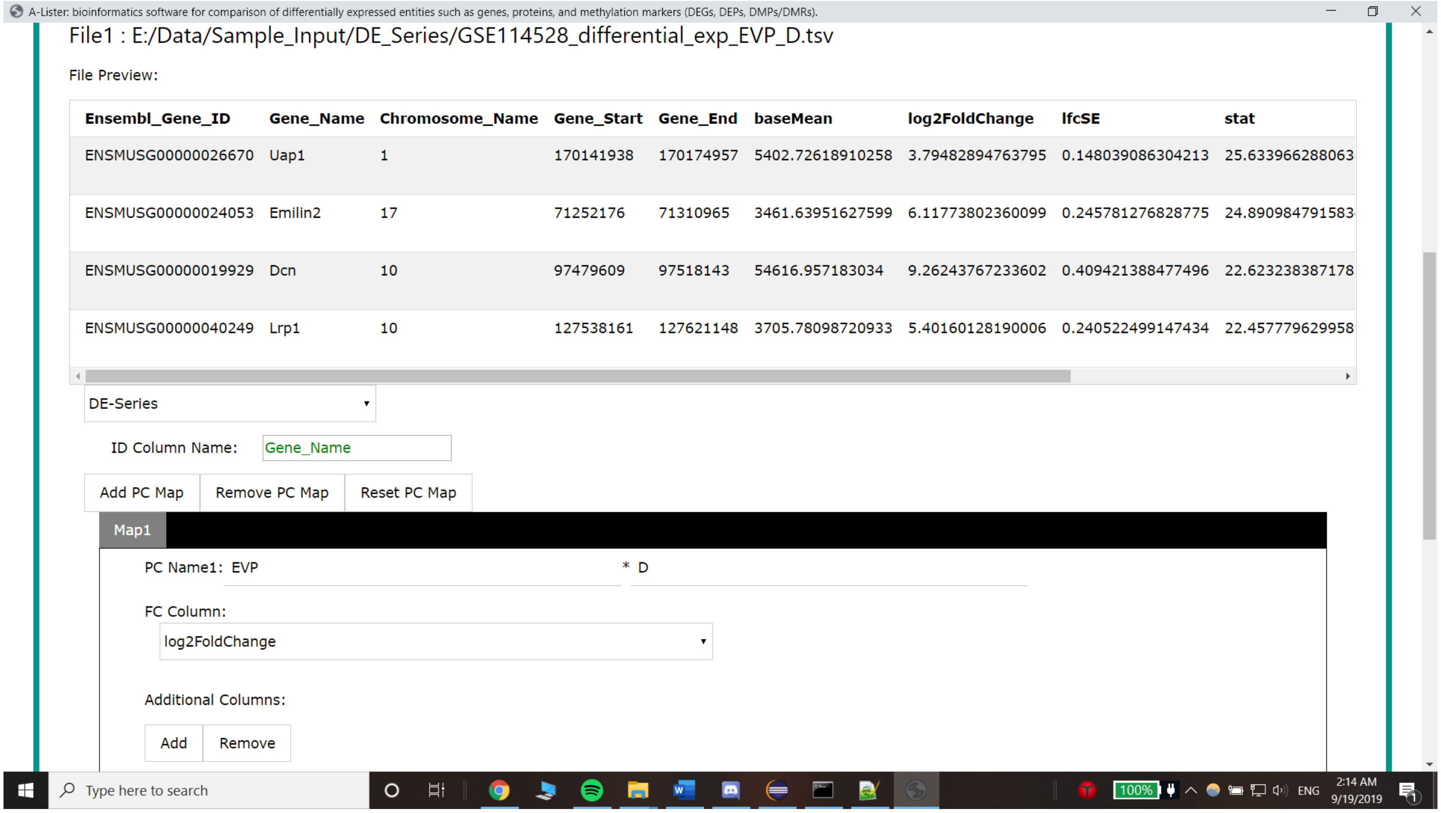

4. Add filters. Herein, we have added fold change, adjusted p-value, and base mean based filters.

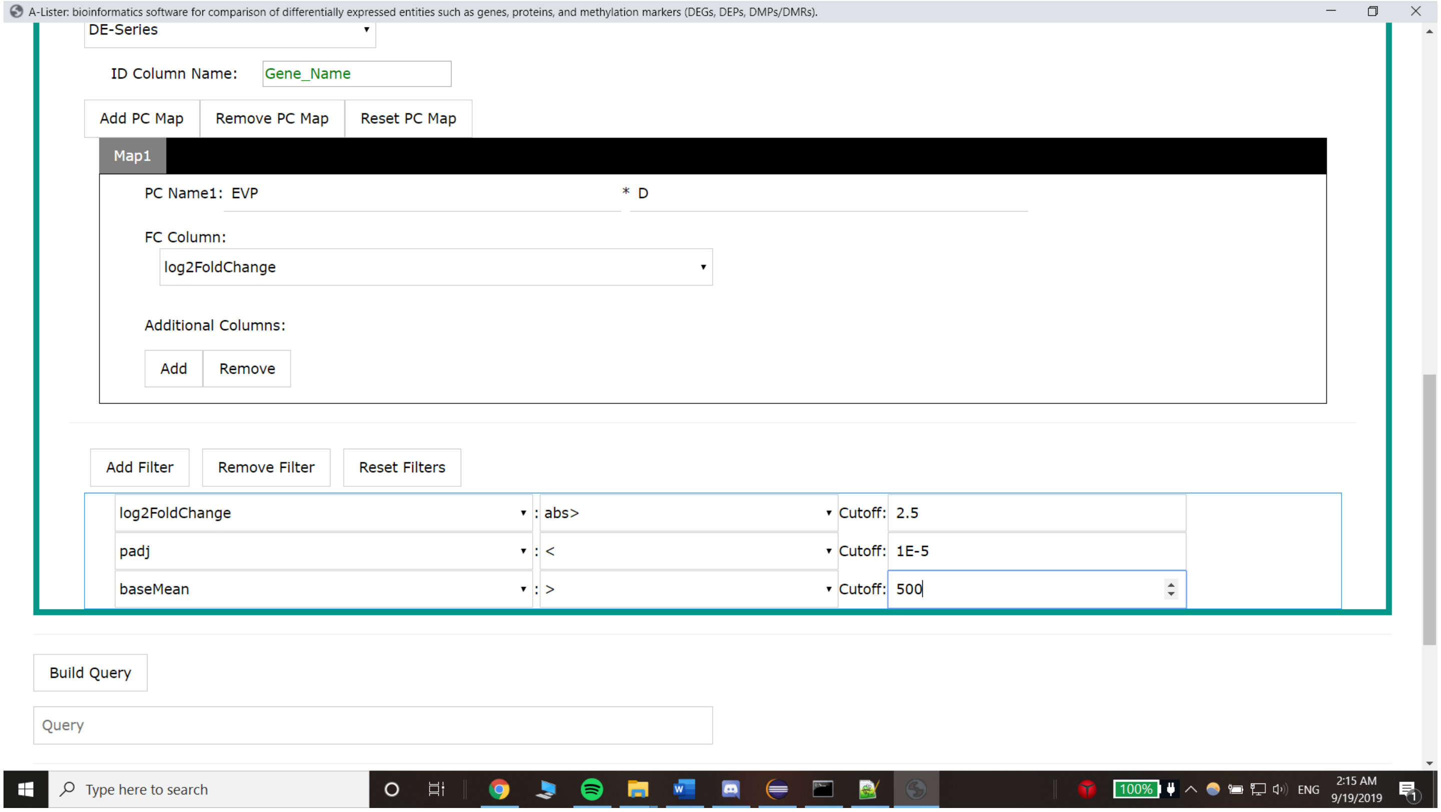

5. Select Sample_Input/DE_Sample/GSE99397_CreNeg-MHCPos_vs_CrePos-MHCNeg.diff file using the "browse" button. Then select the corresponding input file delimiter (tab). Click "Add File" in order to add file to the file selection area. Click on "File2" in the file selection tab bar.
6. Select DE-Sample file type. Verify that the ID, Fold Change, Sample1, and Sample2 column names are correct.

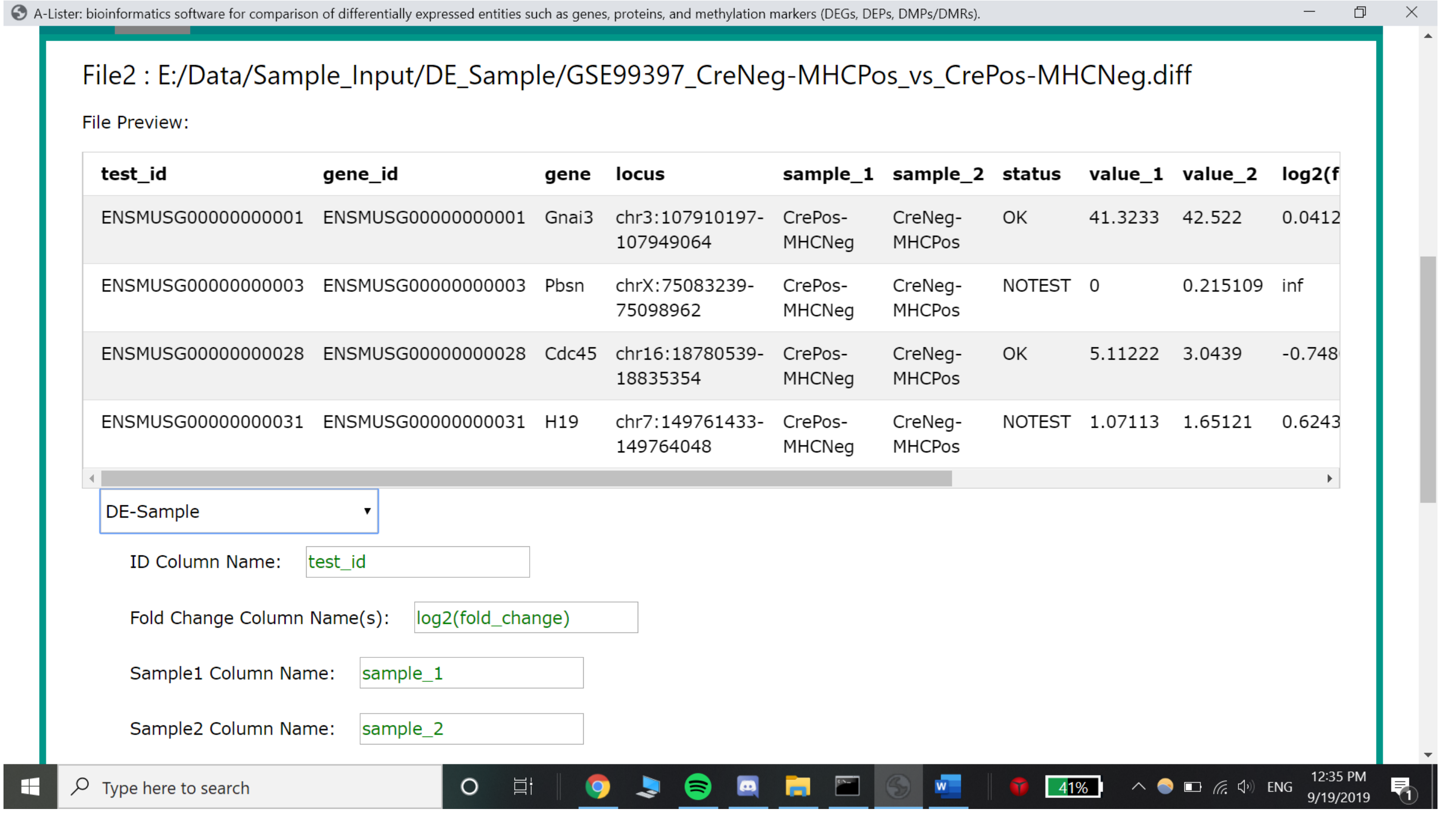

7. Change the value for ID Column Name from test_id to gene. Click on "Build PC Map". Map the file specific sample labels to new label names of your choice. Add filters. Herein, we have added fold change and p-value filters.

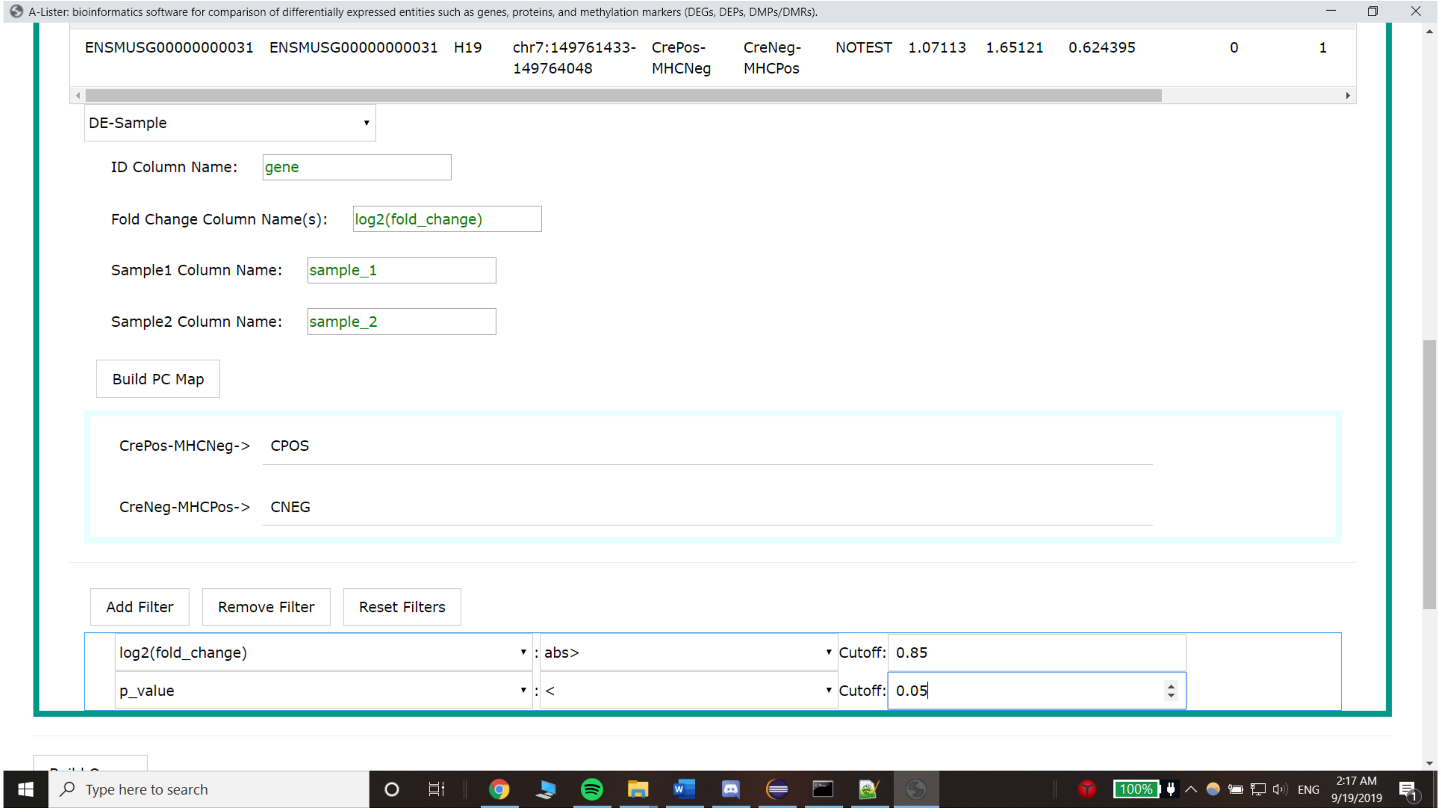

8. Either directly enter the query or click on build query and use the pairwise comparison, set operator, and directionality buttons. You can manually insert parenthesis into the query in order to change order of operations. However, it is not necessary for this simple query.

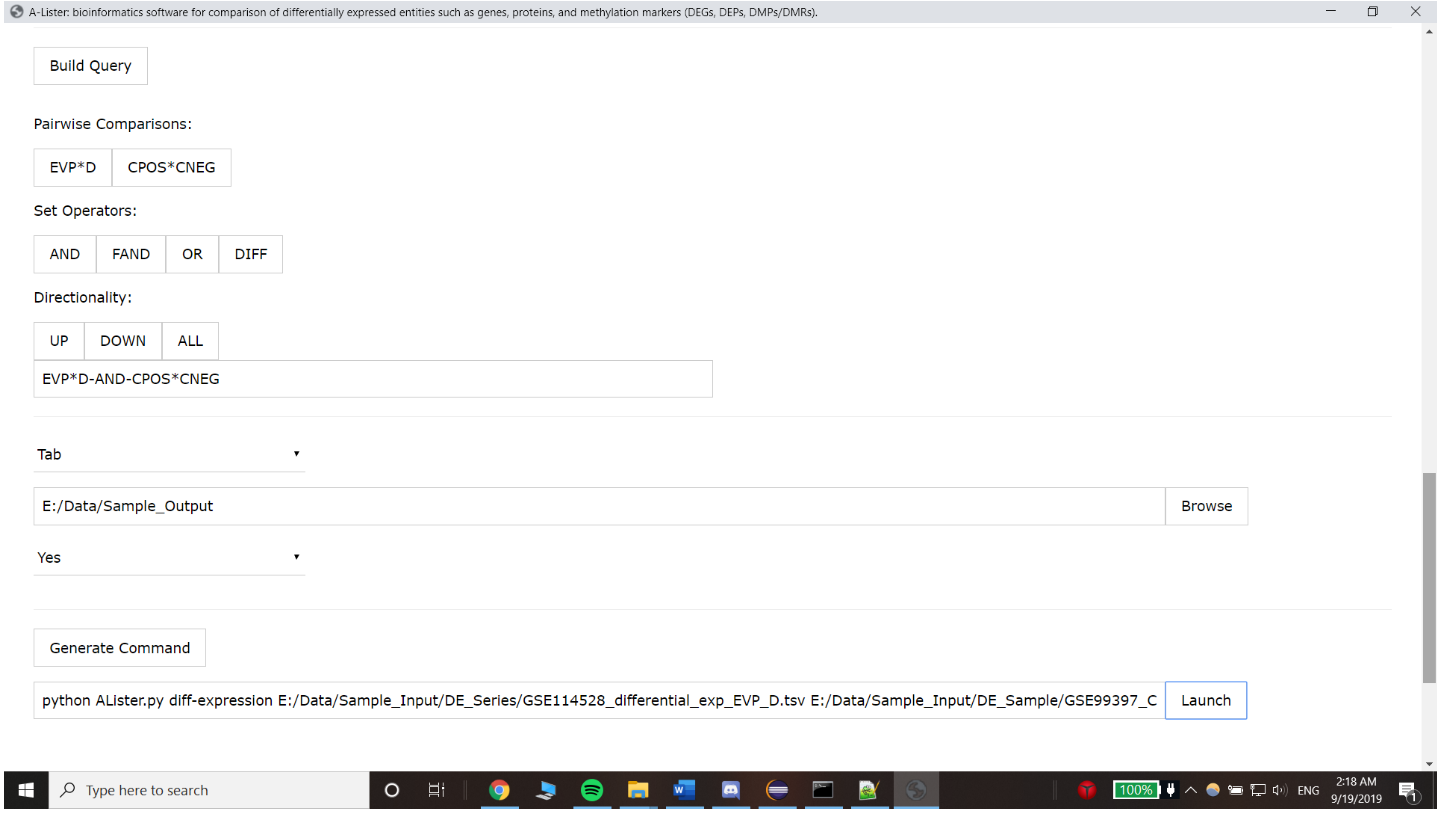

9. Select the output delimiter and output directory.
10. Select whether to run in verbose or silent mode.
11. Click generate command. This generates an appropriate CLI command.

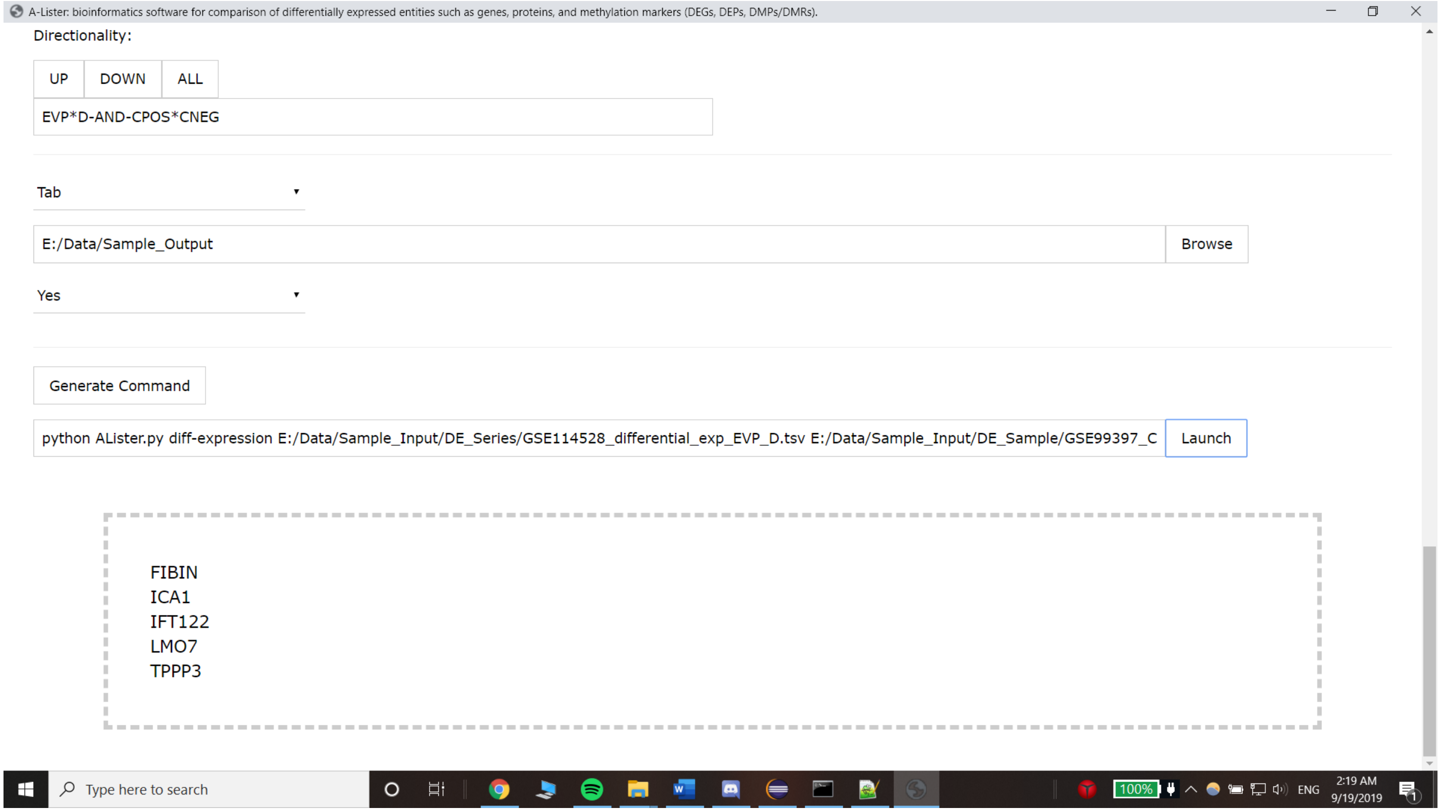

12. You can then launch this command within GUI or by pasting it into command line. Note that the output within the GUI shows the results of a single query only. The complete results can be found in output files.

Command Line Interface Documentation

A-Lister Help

```
$ python AListеr_CLI.py --help
usage:
  ALister_CLI.py command
commands:
  diff-expression Analyze differential expression data.
  name-list       Analyze name list data.

A-Lister v1.1: a differentially expressed entities analysis package. Namely,
A-Lister assists in identification of top genes, proteins, and methylation
markers of interest across multiple pairwise comparisons.

positional arguments:
  {name-list,diff-expression}

optional arguments:
  -h, --help            show this help message and exit
```

Name List Help & Example Commands

```
$ python ALister_CLI.py name-list --help
$ python ALister_CLI.py name-list --examples
```

Differential Expression Help & Example Commands

```
$ python ALister_CLI.py diff-expression --help
$ python ALister_CLI.py diff-expression --examples
```

Future Development Roadmap

1. Pip packaging. User interface update.
2. ID validation and mapping. Genomic types integration.
3. Web interface.

Acknowledgements

The following Gene Expression Omnibus datasets are used as sample and test input within A-Lister: GSE76453, GSE116610, GSE108643, GSE75035, GSE77137, GSE126785, GSE101484, GSE99397, GSE114528. https://www.ncbi.nlm.nih.gov/geo/query/acc.cgi

Authors

Stanislav Listopad and Trina Norden-Krichmar

Apendix

Below are the help menus and partial lists of examples for name-list and diff-expression commands.

A-Lister Name-List Help Menu

```
$ python ALister_CLI.py name-list --help
usage:
  ALister_CLI.py name-list <query> <input-file> [<input-file> ...] [options]
  ALister_CLI.py name-list (-e | --examples)
  ALister_CLI.py name-list (-h | --help)


Analyze name list data.

positional arguments:
  <query>               The non-directional query argument is used to perform set
                        operations on name lists. The group names are derived
                        from the 1st row (column headers) of the delimited text file.
                        The following set operations are permitted: AND, FAND, DIFF, OR.
                        AND = intersect, FAND = fuzzy intersect, DIFF = difference, and
                        OR = union. Additionally, parenthesis can be used to set order of
                        operations. The query argument must be surrounded by quote marks.
                        Example: "(CONTROL-OR-TREATED1)-AND-(TREATED2)"
                        Permitted characters: alphanumeric, (), and -.
  <input-file>          Full path to the input file.

optional arguments:
  -h, --help            show this help message and exit
  -e, --examples        show examples and exit
  -id <input_delimiter> [<input_delimiter> ...]
                        The delimiter used within the input file.
                        List once if all input files use the same delimiter.
                        List for every file - in the same order as that of the
                        input files - if delimiter varies between input files.
                        Options: tab and comma. Default: tab.
  -o <output_directory>
                        The output directory. Default: current working directory.
  -od <output_delimiter>
                        The delimiter used in the result file.
                        Options: tab, comma-row. Default: tab.
  -v                    Make the output more verbose.
```

A-Lister Diff-Expression Help Menu

```
$ python ALister_CLI.py diff-expression --help
usage:
ALister_CLI.py diff-expression <input-file> [<input-file> ...] -pc <pc-mapping> [<pc-mapping> ...] -dq <direct-query>
[options]
ALister_CLI.py diff-expression (-e | --examples)
ALister_CLI.py diff-expression (-h | --help)


Analyze differential expression data.

positional arguments:
<input-file>          Full path to the input file.

required arguments:
-pc <pc-mapping> [<pc-mapping> ...]
                      The pairwise comparison mapping argument is used to indicate
                      the names and the layout of pairwise comparisons within the
                      input file. The formatting of this argument is dependent
                      on the format of the input file (DE-Sample or DE-Series). Within
                      DE-Sample files each entry (row) in a file is already assigned to a
                      pairwise comparison via the labels in sample1 and sample2 columns.
                      A-Lister requires the user to assign each of these file labels new names.
                      For example, assume that there is a DE-Sample file with labels
                      q1, q2, q3, and q4 that represent the control, treated1, treated2,
                      and treated3 conditions. The user would then specify the following
                      mapping: -pc "q1->CNTRL,q2->T1,q3->T2,q4->T3".
                      DE-Series files usually do not specify pairwise comparison names.
                      Therefore, the user must provide their own pairwise comparison names and
                      then map these names to the appropriate columns. For example, assume
                      that there is a DE-Series file with five columns: gene, log2(FC)1, P-value1,
                      log2(FC)2, and P-value2. Assume that this file contains two pairwise
                      comparisons: day0 vs day7 and day7 vs day14. Columns log2(FC)1 and P-value1
                      contain data for day0 vs day7 comparison. Columns log2(FC)2 and P-value2
                      contain data for day7 vs day14 comparison. The user would then specify the
                      following mapping: -pc "d0*d7->log2(FC)1,P-value1;d7*d14->log2(FC)2,P-value2"
                      The star represents versus. Each pairwise comparison must be assigned
                      one unique fold change column (e.g. log2(FC)1 and log2(FC)2 above).
                      For DE-Series files containing a single pairwise comparison it is
                      sufficient to list only the name of the fold change column. For example,
                      assume that the log2(FC)2 and P-value2 columns were removed from the
                      above mentioned file. The appropriate mapping for this new file would
                      be either -pc "day0*day7->log2(FC)1" or -pc "day0*day7->log2(FC)1,P-value1".
                      The pairwise comparison mapping argument must be surrounded by
                      quote marks. Permitted characters: alphanumeric, (), *, ;, -, >.
-dq <direct-query>    The directional query argument is used to perform
                      set operations on pairwise comparisons from differential
                      expression data and to apply directional filtering. The
                      following set operations are permitted: AND, FAND, DIFF, OR.
                      AND = intersection, DIFF = difference, OR = union, FAND =
                      fuzzy intersection. Additionally, parenthesis can be used to
                      nest and to set order of operations. Each pairwise comparison listed
                      in the query can be assigned a direction of UP, DOWN, ALL.
                      All pairwise comparisons have a direction of NONE by default.
                      UP in context of pairwise group comparison q1*q2 means
                      that q2 contains higher expression of this entity than q1.
                      As in fold change - log2(q2/q1) - is a positive value.
                      ALL direction is a special modifier that results in multiple queries.
                      In one query ALL is replaced by UP. In the other query ALL is
                      replaced by DOWN. A query containing N ALL directions is
                      transformed into N^2 queries. Each query is then executed
                      and the results for each query are output into the output files.
                      The names of pairwise comparisons are constructed
                      using the pairwise comparison mapping (refer to -pc option).
                      The query argument must be surrounded by quote marks.
                      Example: "(CNTRL*T1:UP-AND-CNTRL*T2:UP)-DIFF-CNTRL2*T3"
                      Permitted characters: alphanumeric, (), *, :, and -.

optional arguments:
-h, --help            show this help message and exit
```

```
-e, --examples        show examples and exit
-n <id-column> [<id-column> ...]
                      The name of id column within the input files.
                      List once if all input files have the same column name.
                      List for every file - in same order as that of the input files -
                      if column name varies between input files. The id column argument
                      must be surrounded by quote marks. Default: Id.
-fc <fc-column> [<fc-column> ...]
                      The names of fold change columns within the input files.
                      List once if all input files have the same column names.
                      List for every file - in same order as that of the input files -
                      if column names vary between input files. Multiple fold change
                      columns can be listed for DE-Series files. When listing
                      multiple fold change columns for a DE-Series file delimit
                      the column names with commas. The fold change column argument
                      must be surrounded by quote marks.
                      Default: log2(fold_change).
-s1 <sample1-column> [<sample1-column> ...]
                      The name of sample1 column within the input files.
                      List once if all input files have the same column name.
                      List for every file - in same order as that of the input
                      files - if column name varies between input files. For DE-Series
                      files the sample1 column name must be listed as "NONE".
                      The sample1 column argument must be surrounded by
                      quote marks. Default: NONE.
-s2 <sample2-column> [<sample2-column> ...]
                      The name of sample2 column within the input files.
                      List once if all input files have the same column name.
                      List for every file - in same order as that of the input
                      files - if column name varies between input files. For DE-Series
                      files the sample2 column name must be listed as "NONE".
                      The sample2 column argument must be surrounded by
                      quote marks. Default: NONE.
-f <filter-by-column> [<filter-by-column> ...]
                      The names of columns by which to filter the files alongside
                      the respective comparison operators and comparison constant values.
                      List once if you would like to filter all files the same way.
                      List for every file - in same order as that of the input files -
                      otherwise. If you would like to filter some, but not all of the
                      files, write "NONE" for the files that you do not want to be filtered.
                      The following comparison operators are permitted: lt (a<b),
                      le (a<=b), gt (a>b), ge (a>=b), eq (a==b), ne (a!=b),
                      alt (|a|<b), ale (|a|<=b), agt (|a|>b), age (|a|>=b),
                      aeq (|a|==b), ane (|a|!=b).
                      This argument must be surrounded by quote marks.
                      Example: "log2(fold_change):agt1.2,p_value:lt0.05,q_value:lt0.05."
-id <input_delimiter> [<input_delimiter> ...]
                      The delimiter used within the input file.
                      List once if all input files use the same delimiter.
                      List for every file - in the same order as that of the
                      input files - if delimiter varies between input files.
                      Options: tab, comma, colon, semicolon, space.
                      Default: tab.
-o <output_directory>
                      The output directory. Default: current working directory.
-od <output_delimiter>
                      The delimiter/format used in the result file.
                      Options: tab, comma-row. Default: tab.
-v                    Make the output more verbose.
```

A-Lister Name-List Examples

```
$ python ALister_CLI.py name-list --examples
# Example Name List 1:
# One name-list file. Three groups: Control, Treated1, and Treated2.
# Query is an intersection of two groups.
```

```
python ALister_CLI.py name-list "Treated1-AND-Treated2" E:/Data/Sample_Input/Name_List/names_comma.txt
-id comma -o E:/Data/Sample_Output/Name_List/Example1 -od tab -v

# Example Name List 2:
# Two name-list files. Complex query across groups from both files.

python ALister_CLI.py name-list "Set1-DIFF-(Control-OR-(Treated1-AND-Treated2))" E:/Data/Sample_Input/Name_List/fileA.txt
E:/Data/Sample_Input/Name_List/names_comma.txt -id tab comma -o E:/Data/Sample_OutputName_List/Example2 -od comma-row -v
```

A-Lister Diff-Expression Examples

```
$ python ALister_CLI.py diff-expression --examples

Example Set 1 - DE-Sample Files Only:
-------------------------------------
# Exercise in Obese vs Lean Individuals (DE Example 3):
# One Cuffdiff file with four condition labels (LeanPre,LeanPost,OvobPre,OvobPost).
# Filter file with several attributes. Launch an ALL direction query (4 direction patterns - UU,UD,DU,DD).

python ALister_CLI.py diff-expression E:/Data/Sample_Input/DE_Sample/GSE108643_Cuffdiff.txt
-pc "LeanPre->LPE,LeanPost->LPO,OvobPre->OPE,OvobPost->OPO" -dq "LPE*LPO:ALL-AND-OPE*OPO:ALL"
-o E:/Data/Sample_Output/DE/Example3 -f "log2(fold_change):agt1.0,q_value:lt0.05,value_1:gt1.0,value_2:gt1.0"
-v -s1 "sample_1" -s2 "sample_2" -n "gene"

Example Set 2 - DE-Series files only:
-------------------------------------
# Effect of Oxygen Saturation on Stem Cells (DE Example 7):
# Three DESeq2 files with one pairwise comparison in each file.
# Filter the pairwise comparisons across both files according to fold change.
# Execute a complex query across all pairwise comparisons.

python ALister_CLI.py diff-expression E:/Data/Sample_Input/DE_Series/GSE126785_M2.txt
E:/Data/Sample_Input/DE_Series/GSE126785_M4.txt E:/Data/Sample_Input/DE_Series/GSE126785_M5.txt
-pc "M2Low*M2High->3.log2(FC)" "M4Low*M4High->3.log2(FC)" "M5Low*M5High->3.log2(FC)"
-dq "M2Low*M2High-DIFF-(M5Low*M5High-OR-M4Low*M4High)" -o E:/Data/Sample_Output/DE/Example7 -n "1.GeneID"
-f "3.log2(FC):agt1.0" -fc "3.log2(FC)" -v

Example Set 3 - DE-Series and DE-Sample files:
---------------------------------------------
Melanoma in Mice (DE Example 8):
# One Cuffdiff and one DESeq2 file with one pairwise comparison in each file.
# Filter the Cuffdiff file according to fold change and p-value.
# Filter the DESeq2 file according to base mean, fold change, and adjusted p-value.
# Execute a query across EVP*D and CPOS*CNEG pairwise comparisons.

python ALister_CLI.py diff-expression E:/Data/Sample_Input/DE_Series/GSE114528_differential_exp_EVP_D.tsv
E:/Data/Sample_Input/DE_Sample/GSE99397_CreNeg-MHCPos_vs_CrePos-MHCNeg.diff -pc "EVP*D->log2FoldChange"
"CrePos-MHCNeg->CPOS,CreNeg-MHCPos->CNEG" -dq "EVP*D-AND-CPOS*CNEG" -fc "log2FoldChange" "log2(fold_change)"
-o E:/Data/Sample_Output/DE/Example8 -od comma-row -f "padj:lt1E-5,log2FoldChange:agt2.5,baseMean:gt500"
"log2(fold_change):agt0.85,p_value:lt0.05" -v -s1 "NONE" "sample_1" -s2 "NONE" "sample_2" -n "Gene_Name" "gene"
```
