# Supplementary material for: A-Lister: a tool for analysis of differentially expressed omics entities across multiple pairwise comparisons
Source: BMC Bioinformatics. 2019 Nov 19;20:595. doi: 10.1186/s12859-019-3121-x (PMC6862834; doi:10.1186/s12859-019-3121-x)
Supplement: Supplementary file 9 — Additional file 9. A-Lister source code. [file 12859_2019_3121_MOESM9_ESM.zip › A-Lister-master/Web/A_Lister_Front.html]

A-Lister: bioinformatics software for comparison of differentially expressed entities such as genes, proteins, and methylation markers
(DEGs, DEPs, DMPs/DMRs).


Close ×
Main Menu
Documentation

☰

# A-Lister

Select the comparison mode:

Differential Expression Mode: Filter and compare entities (genes, proteins, methylation markers, etc.) across pairwise comparisons.

Name List Mode: Compare multiple lists of names.

Browse

Input Delimiter
Tab
Comma
Colon
Semicolon
Space

Add File

Remove File

Reset Files

---

Build Query


---


Output Delimiter
Tab
Comma-Row

Browse

Verbose
No
Yes


---

Generate Command

Launch

Browse

Input Delimiter
Tab
Comma

Add File

Remove File

Reset Files

---

Build Query


---


Output Delimiter
Tab
Comma-Row

Browse

Verbose
No
Yes


---

Generate Command

Launch

## Full documentation can be found on A-Lister GitHub page:

https://github.com/staslist/A-Lister
