# Supplementary figures and images for: A-Lister: a tool for analysis of differentially expressed omics entities across multiple pairwise comparisons
Source: BMC Bioinformatics. 2019 Nov 19;20:595. doi: 10.1186/s12859-019-3121-x (PMC6862834; doi:10.1186/s12859-019-3121-x)

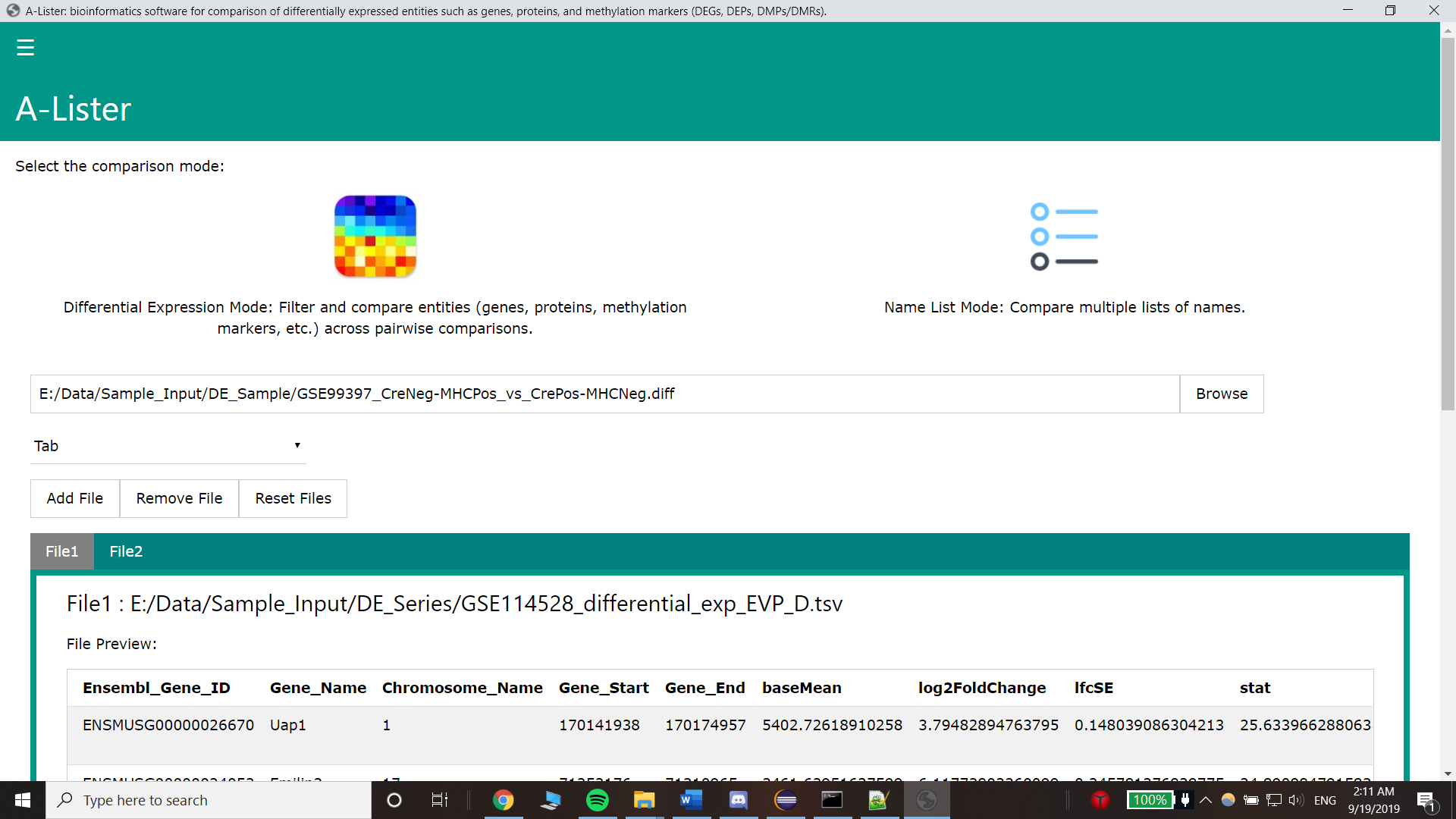

Supplement: Supplementary file 9 — Additional file 9. A-Lister source code. [file 12859_2019_3121_MOESM9_ESM.zip › A-Lister-master/Images/DiffExpressionGUI1.png]

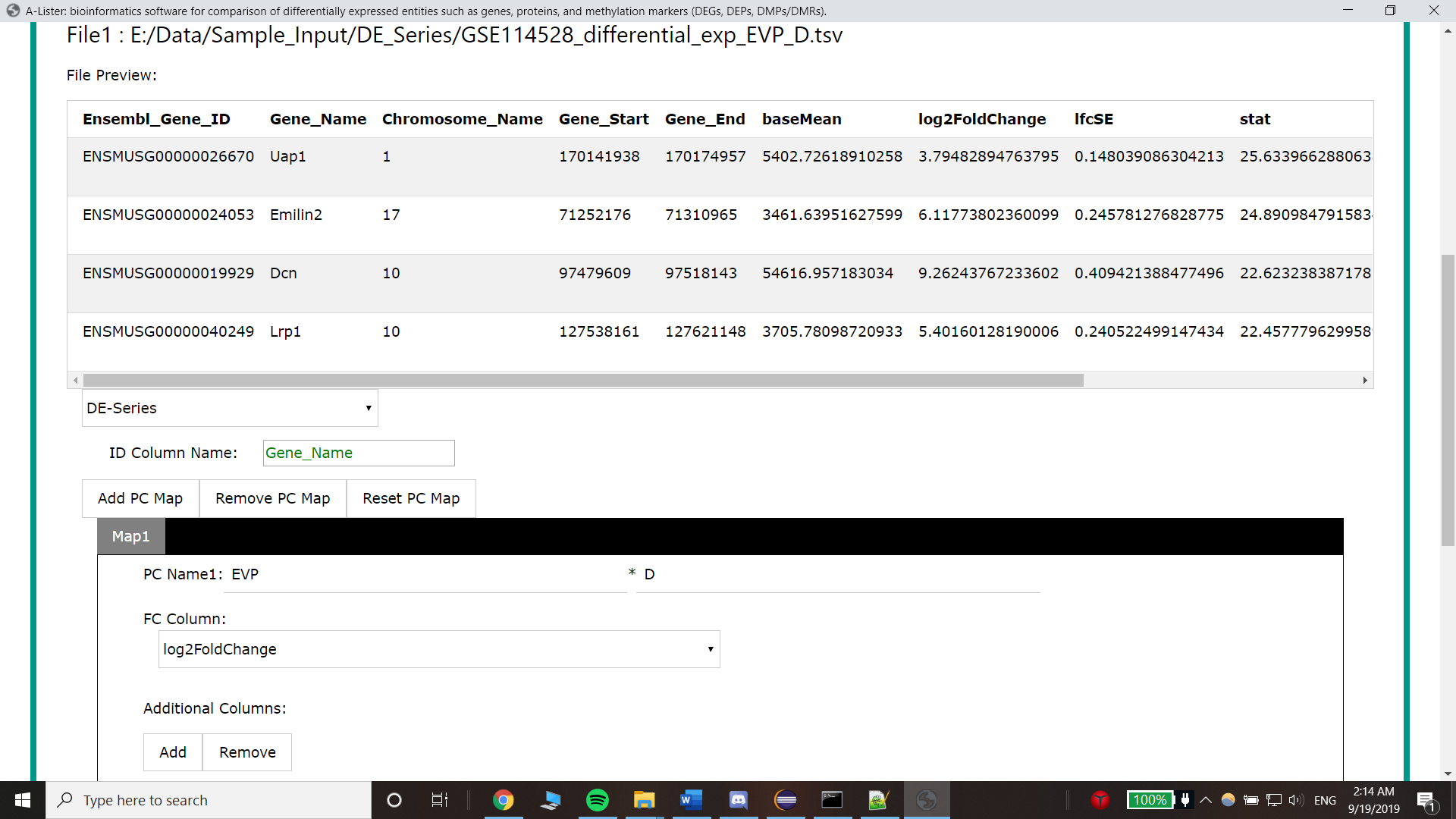

Supplement: Supplementary file 9 — Additional file 9. A-Lister source code. [file 12859_2019_3121_MOESM9_ESM.zip › A-Lister-master/Images/DiffExpressionGUI2.png]

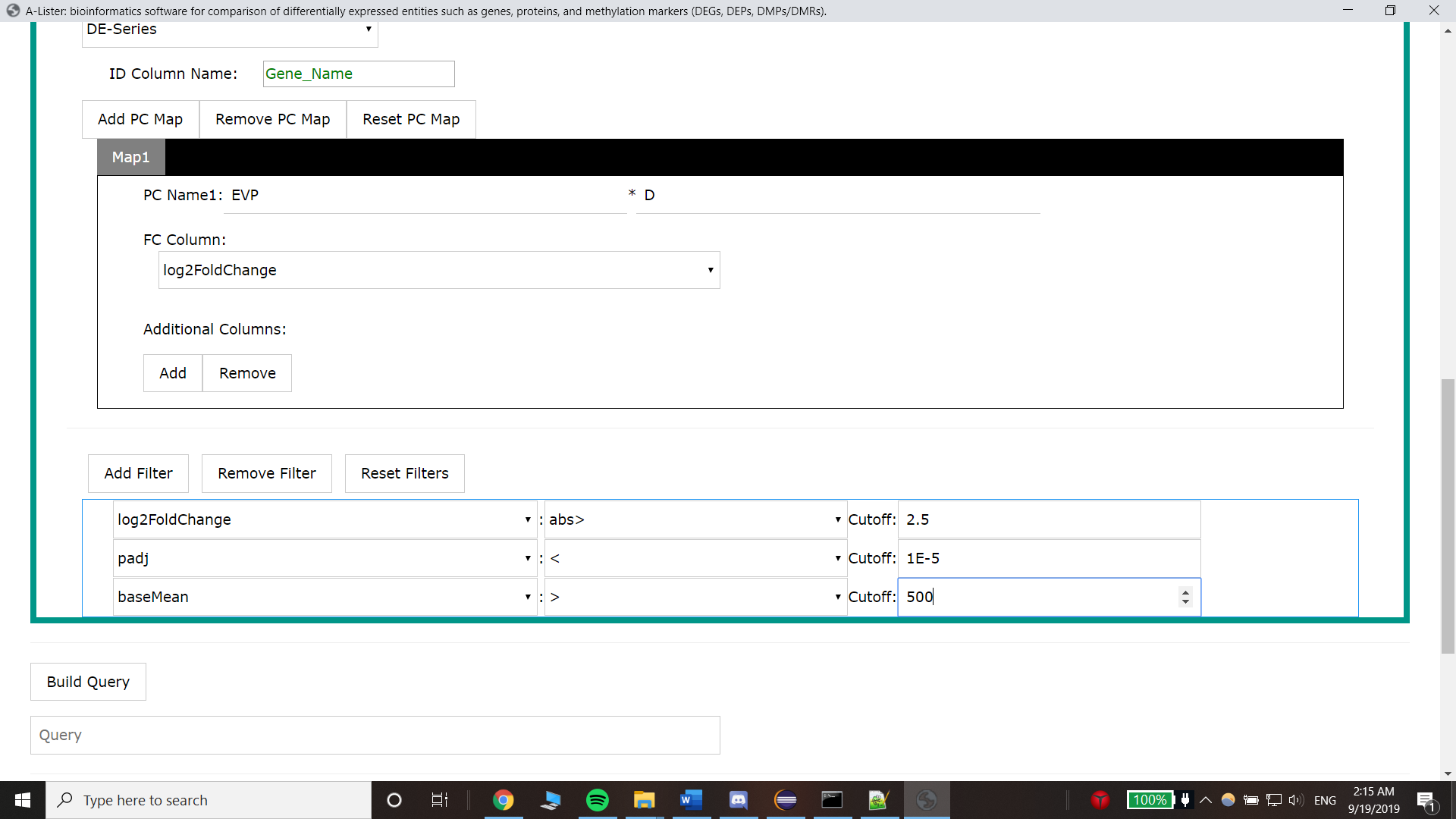

Supplement: Supplementary file 9 — Additional file 9. A-Lister source code. [file 12859_2019_3121_MOESM9_ESM.zip › A-Lister-master/Images/DiffExpressionGUI3.png]

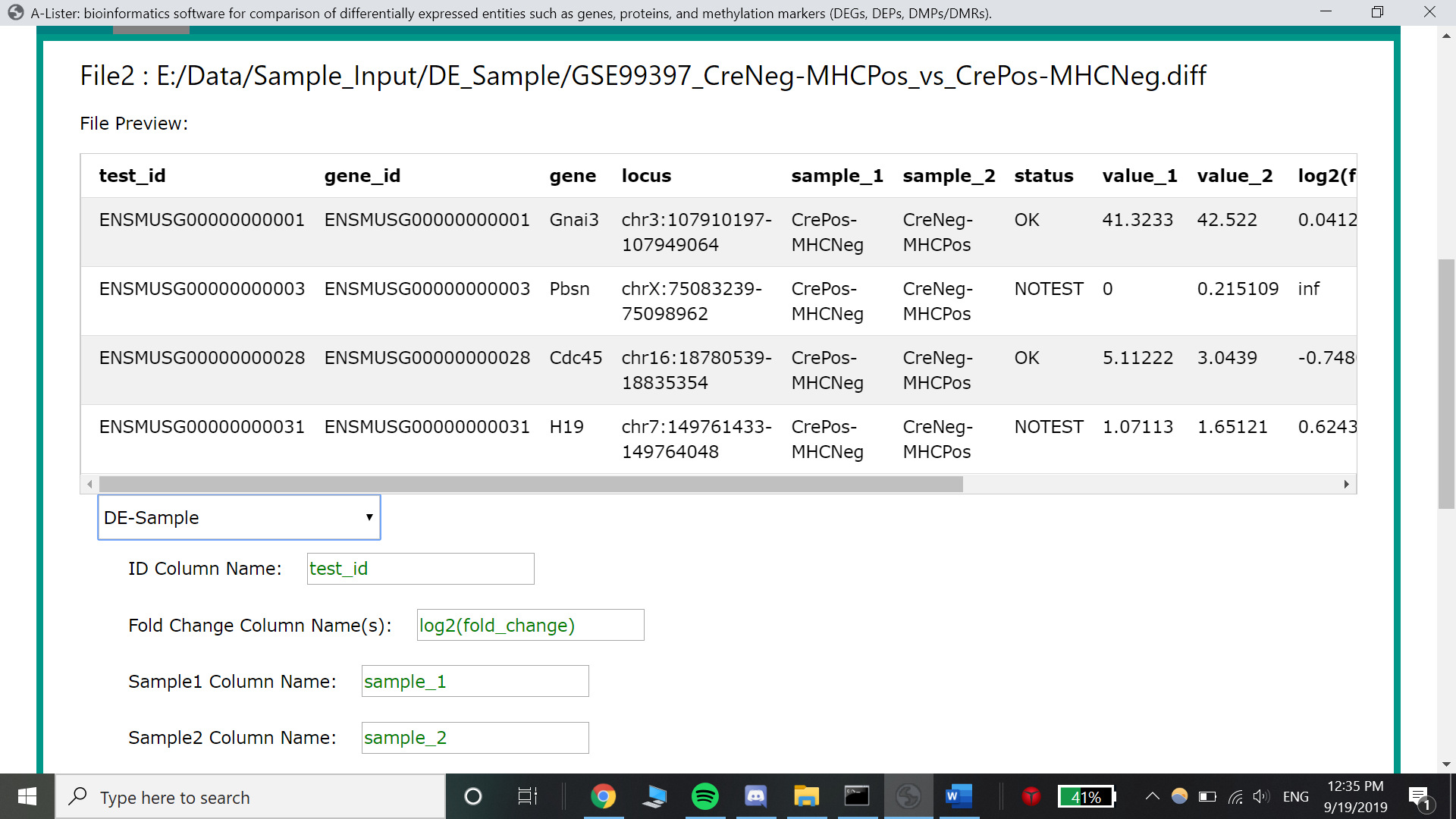

Supplement: Supplementary file 9 — Additional file 9. A-Lister source code. [file 12859_2019_3121_MOESM9_ESM.zip › A-Lister-master/Images/DiffExpressionGUI4.png]

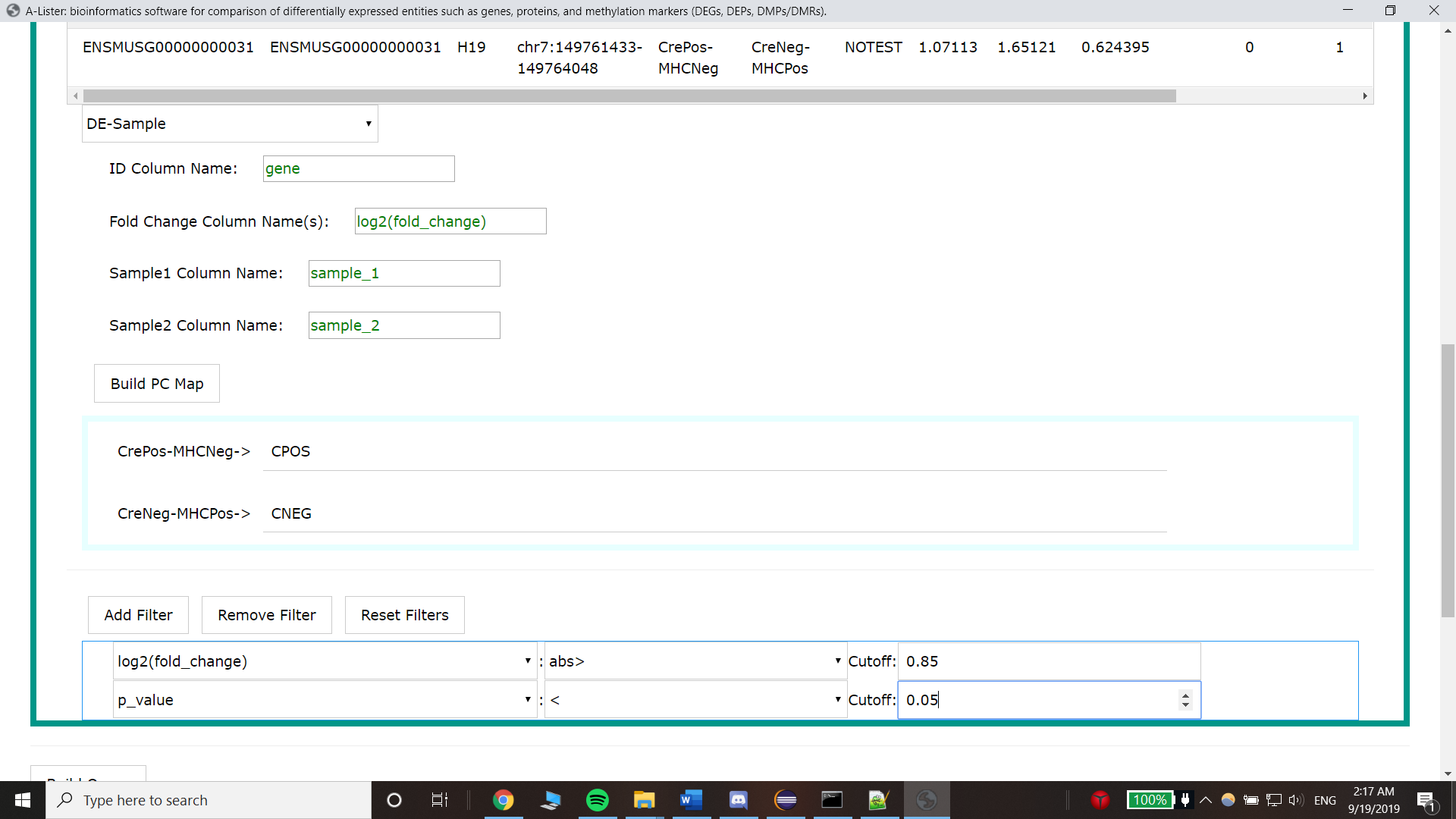

Supplement: Supplementary file 9 — Additional file 9. A-Lister source code. [file 12859_2019_3121_MOESM9_ESM.zip › A-Lister-master/Images/DiffExpressionGUI5.png]

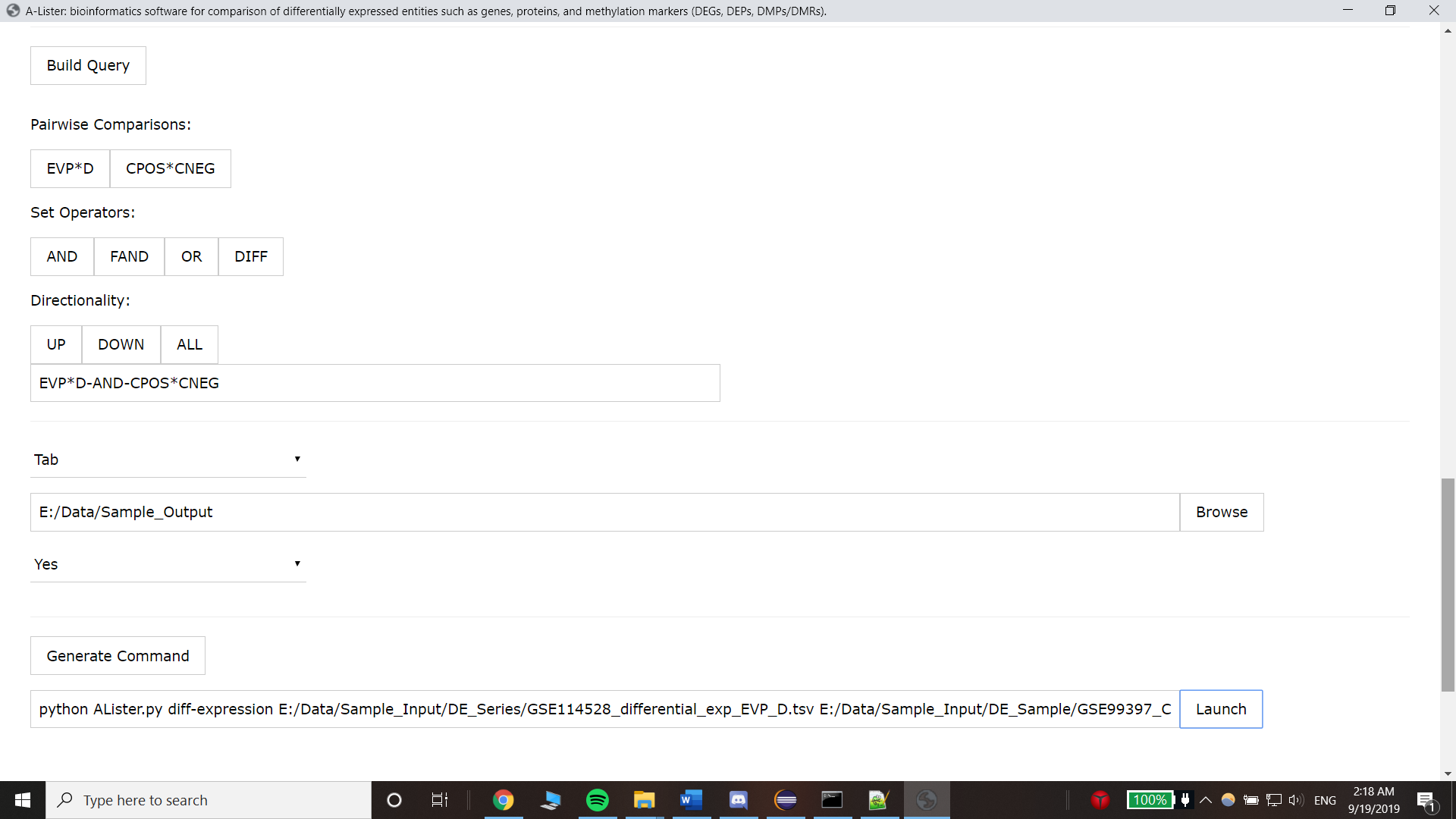

Supplement: Supplementary file 9 — Additional file 9. A-Lister source code. [file 12859_2019_3121_MOESM9_ESM.zip › A-Lister-master/Images/DiffExpressionGUI6.png]

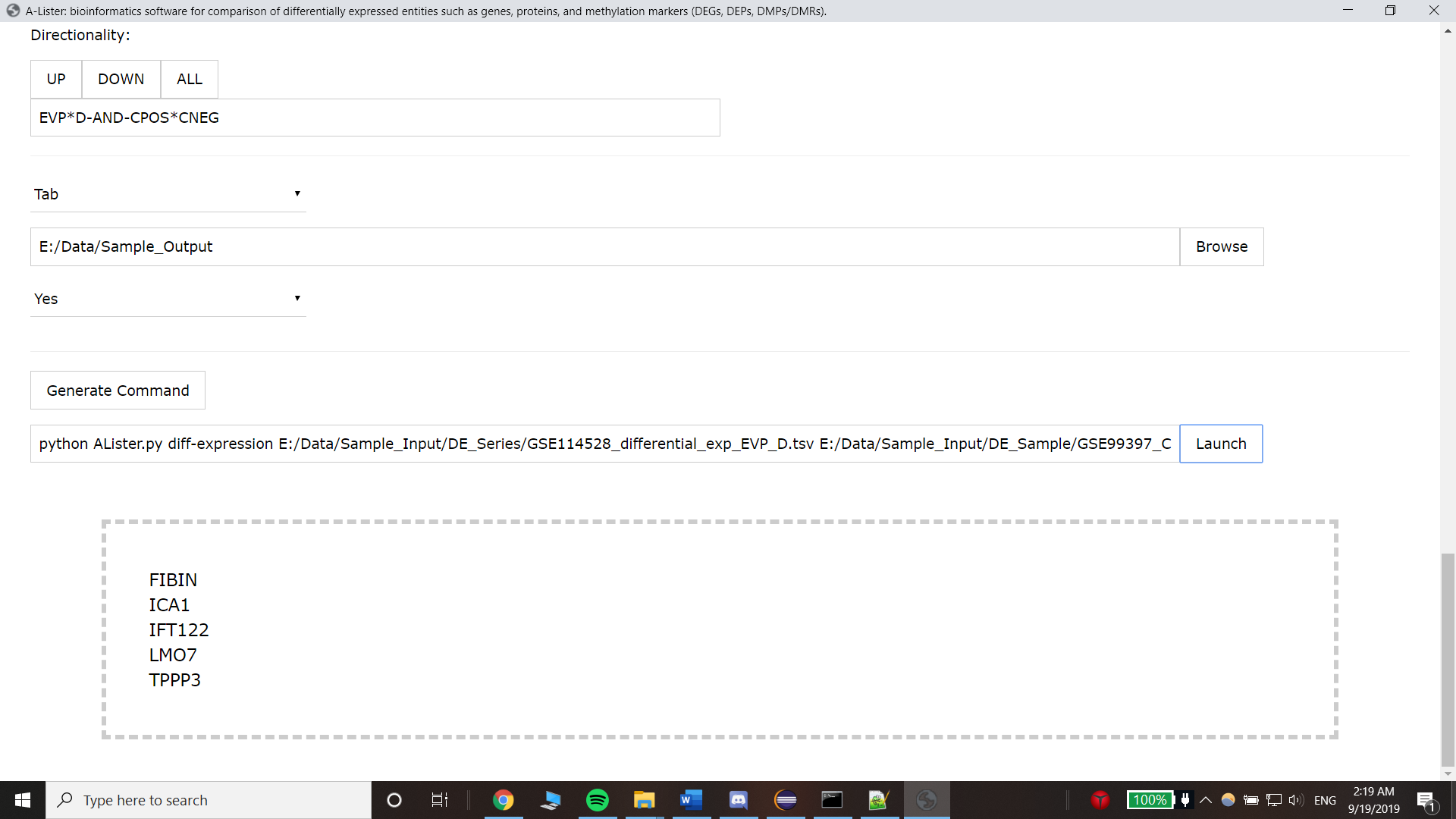

Supplement: Supplementary file 9 — Additional file 9. A-Lister source code. [file 12859_2019_3121_MOESM9_ESM.zip › A-Lister-master/Images/DiffExpressionGUI7.png]

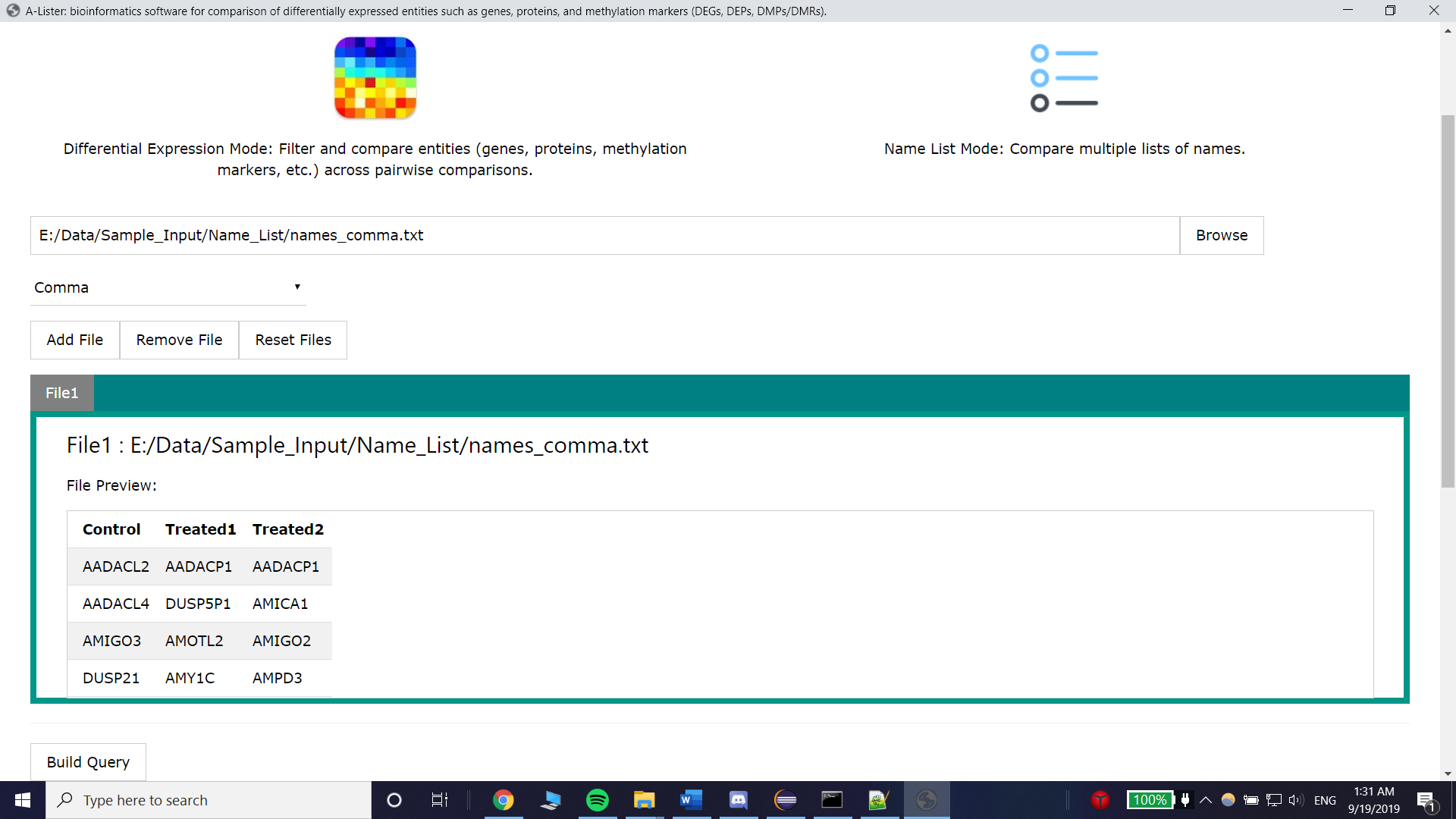

Supplement: Supplementary file 9 — Additional file 9. A-Lister source code. [file 12859_2019_3121_MOESM9_ESM.zip › A-Lister-master/Images/NameListGUI1.png]

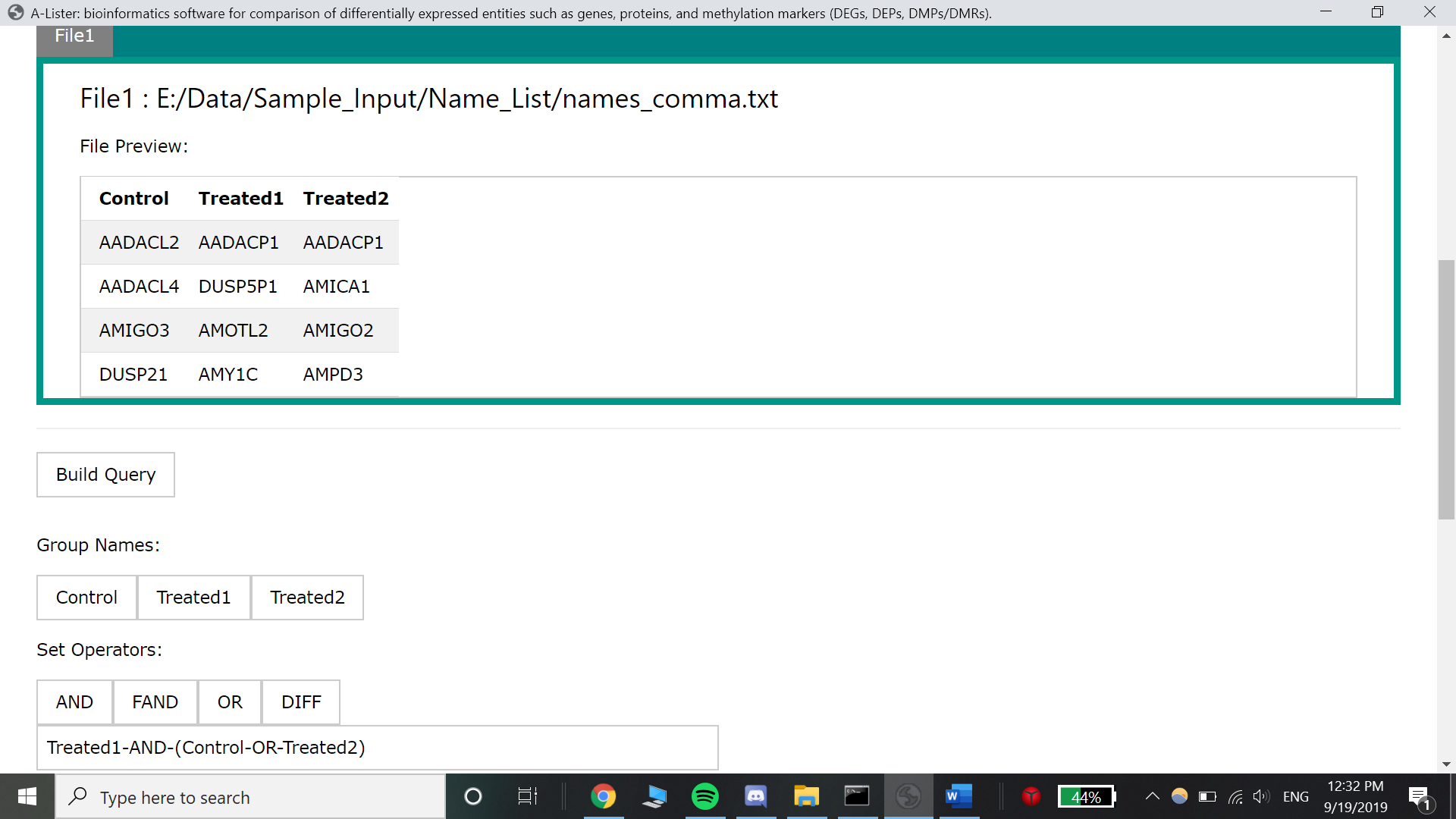

Supplement: Supplementary file 9 — Additional file 9. A-Lister source code. [file 12859_2019_3121_MOESM9_ESM.zip › A-Lister-master/Images/NameListGUI2.png]

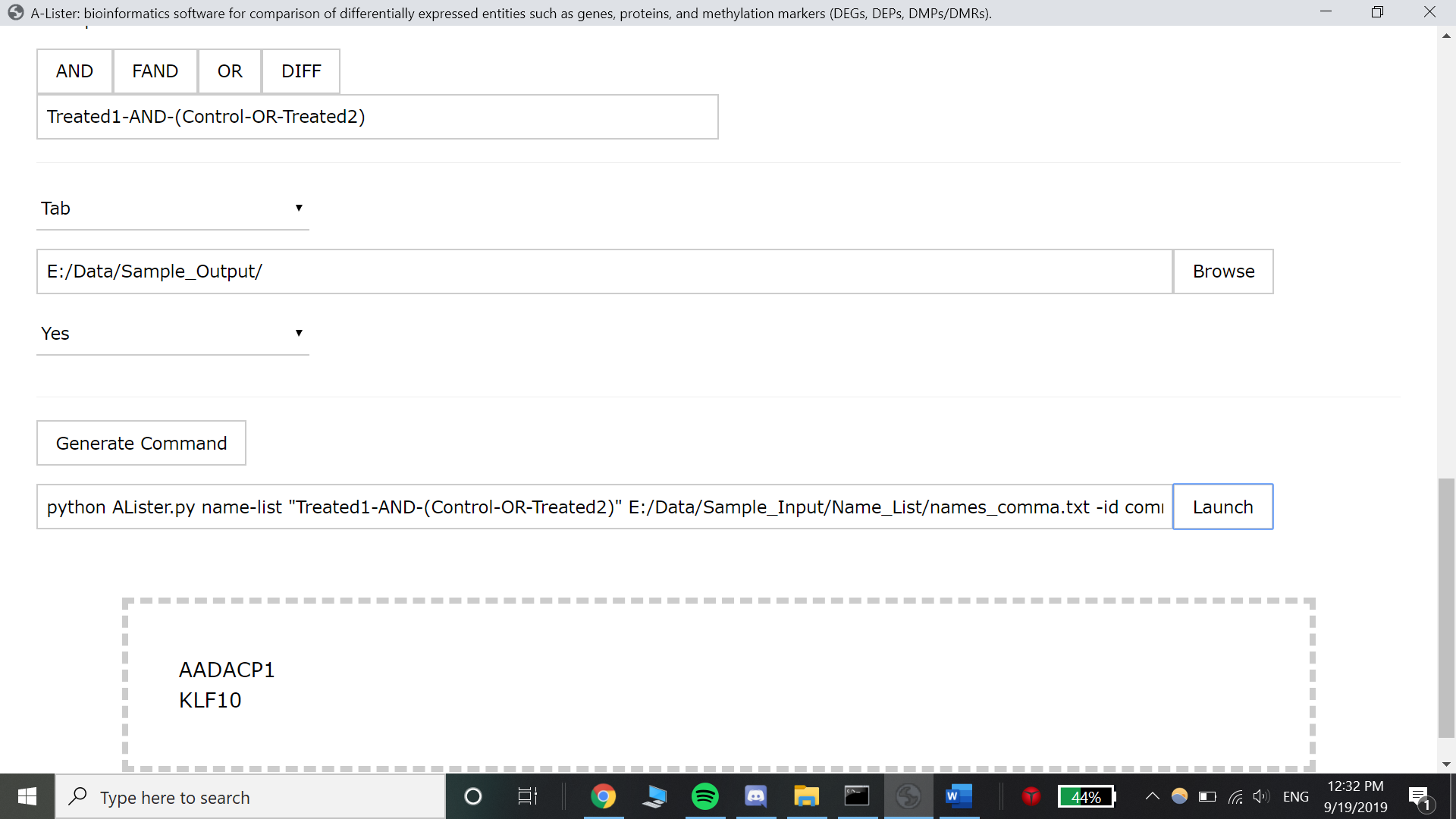

Supplement: Supplementary file 9 — Additional file 9. A-Lister source code. [file 12859_2019_3121_MOESM9_ESM.zip › A-Lister-master/Images/NameListGUI3.png]

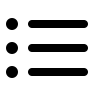

Supplement: Supplementary file 9 — Additional file 9. A-Lister source code. [file 12859_2019_3121_MOESM9_ESM.zip › A-Lister-master/Web/List1.png]

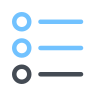

Supplement: Supplementary file 9 — Additional file 9. A-Lister source code. [file 12859_2019_3121_MOESM9_ESM.zip › A-Lister-master/Web/List2.png]

Name List:


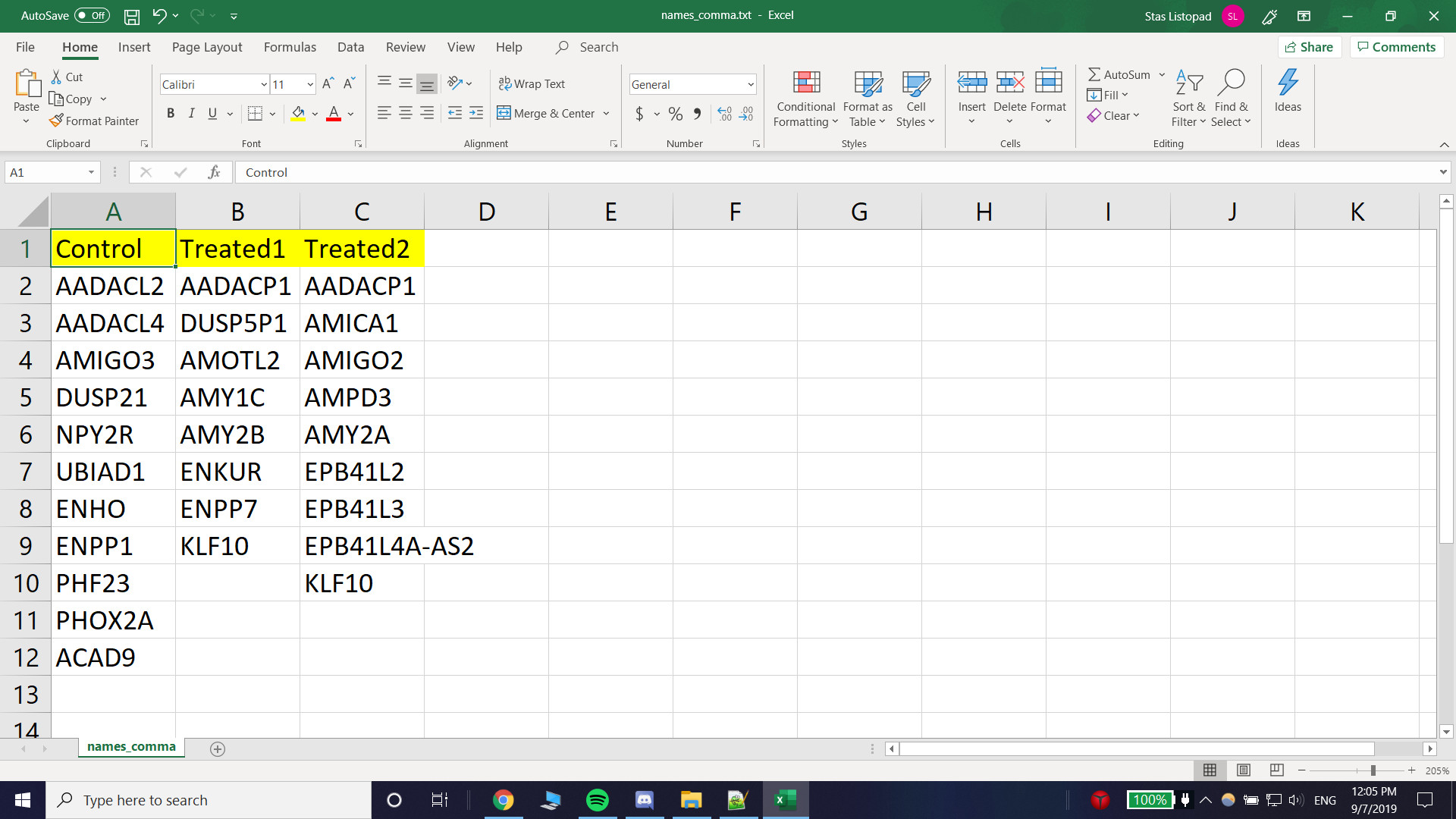


DE Sample:


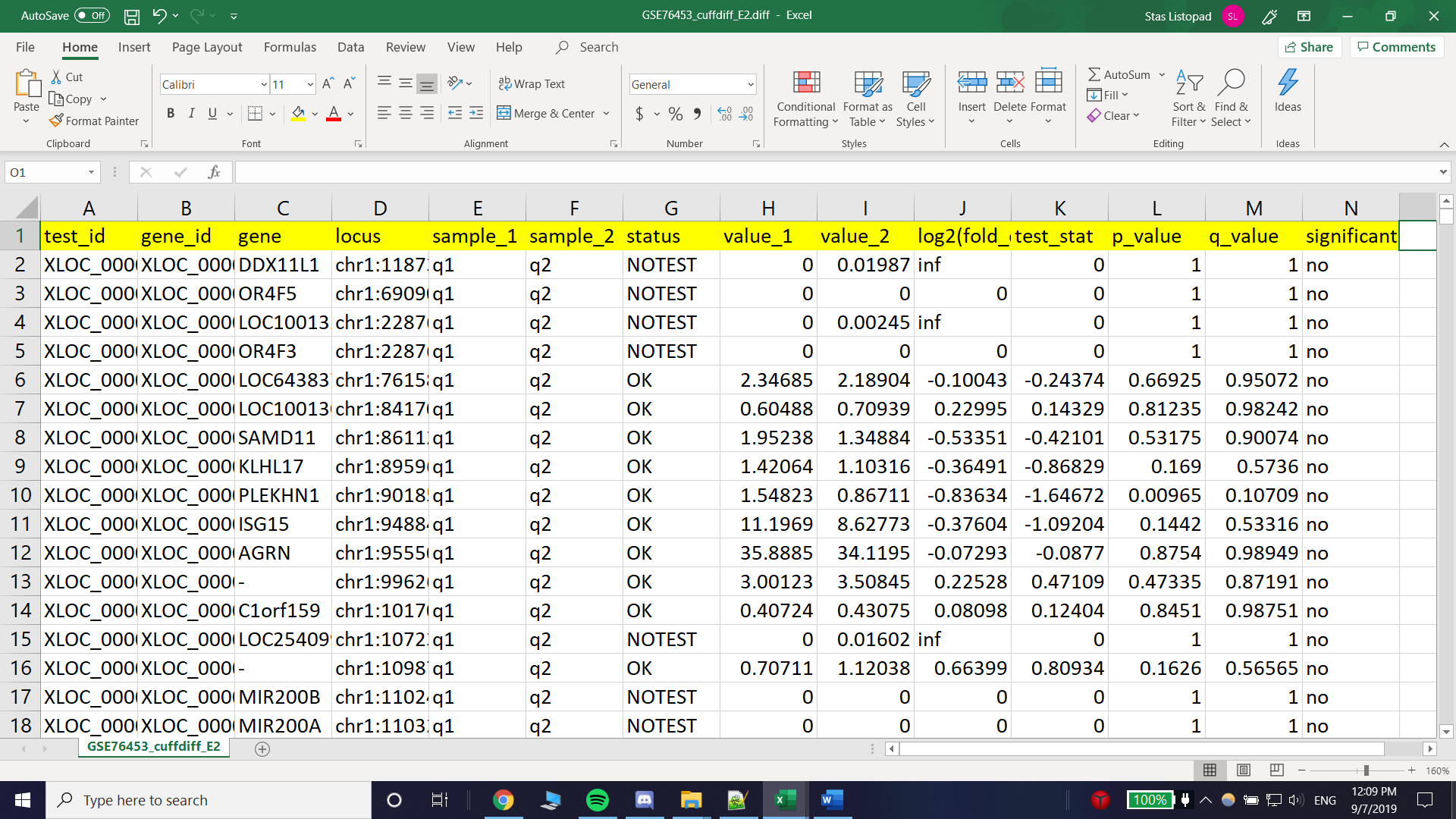


DE Series:


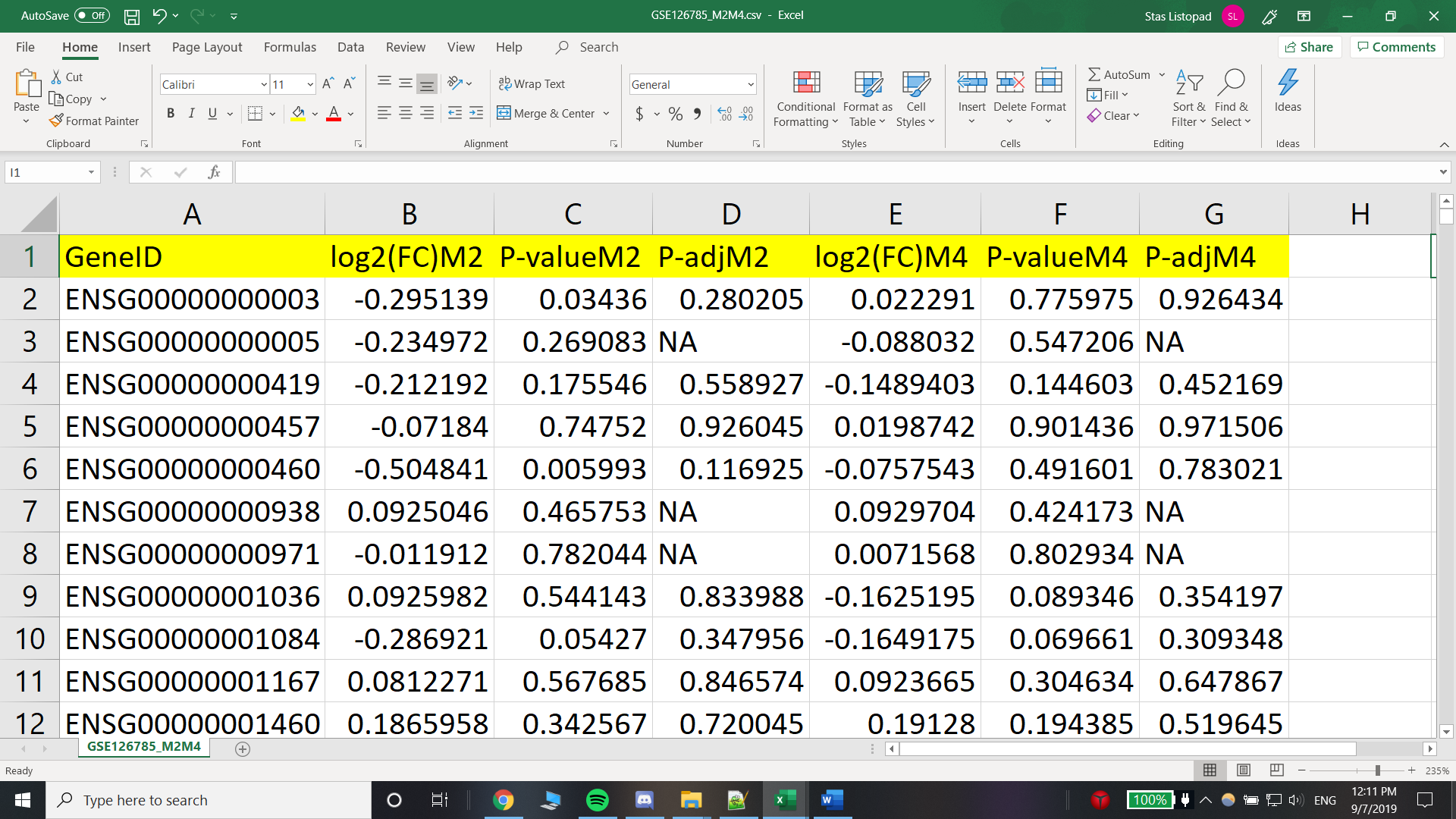

Supplement: Supplementary file 9 — Additional file 9. A-Lister source code. [file 12859_2019_3121_MOESM9_ESM.zip › A-Lister-master/Web/Screenshots.docx]

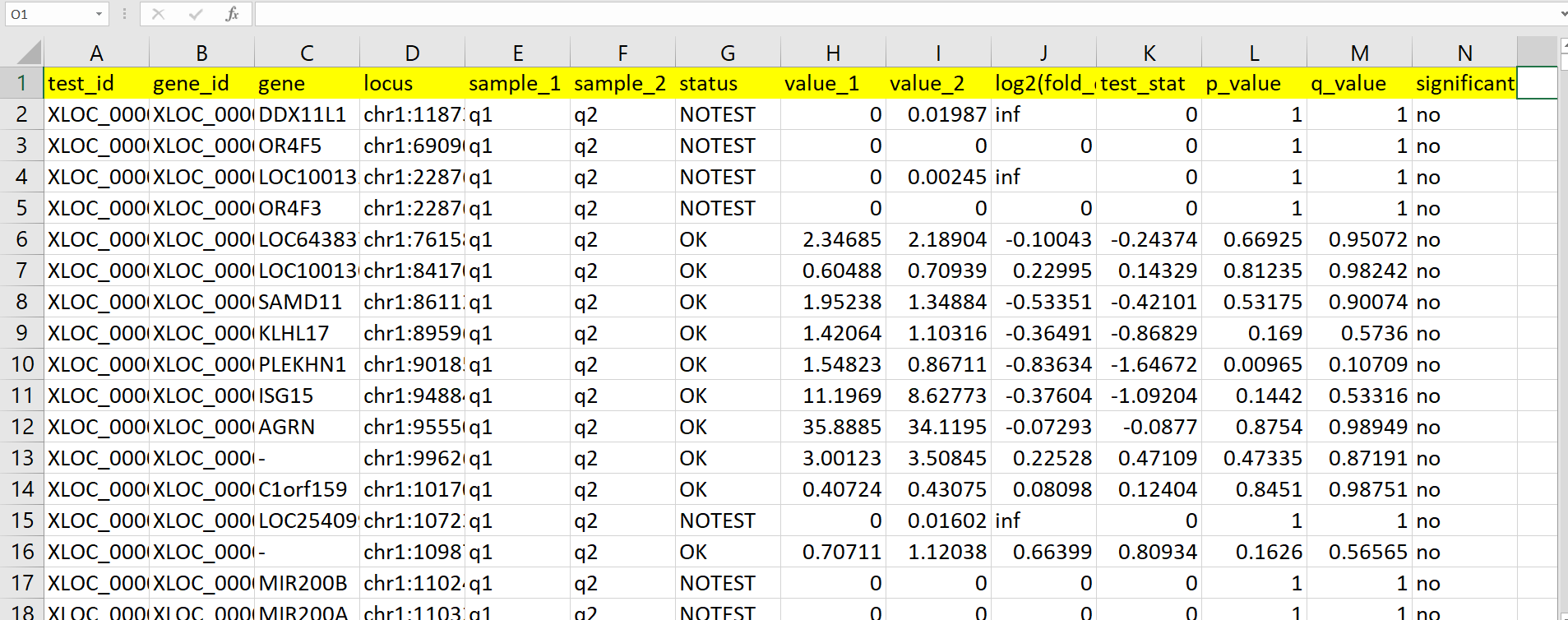

Supplement: Supplementary file 9 — Additional file 9. A-Lister source code. [file 12859_2019_3121_MOESM9_ESM.zip › A-Lister-master/Web/de_sample_file.png]

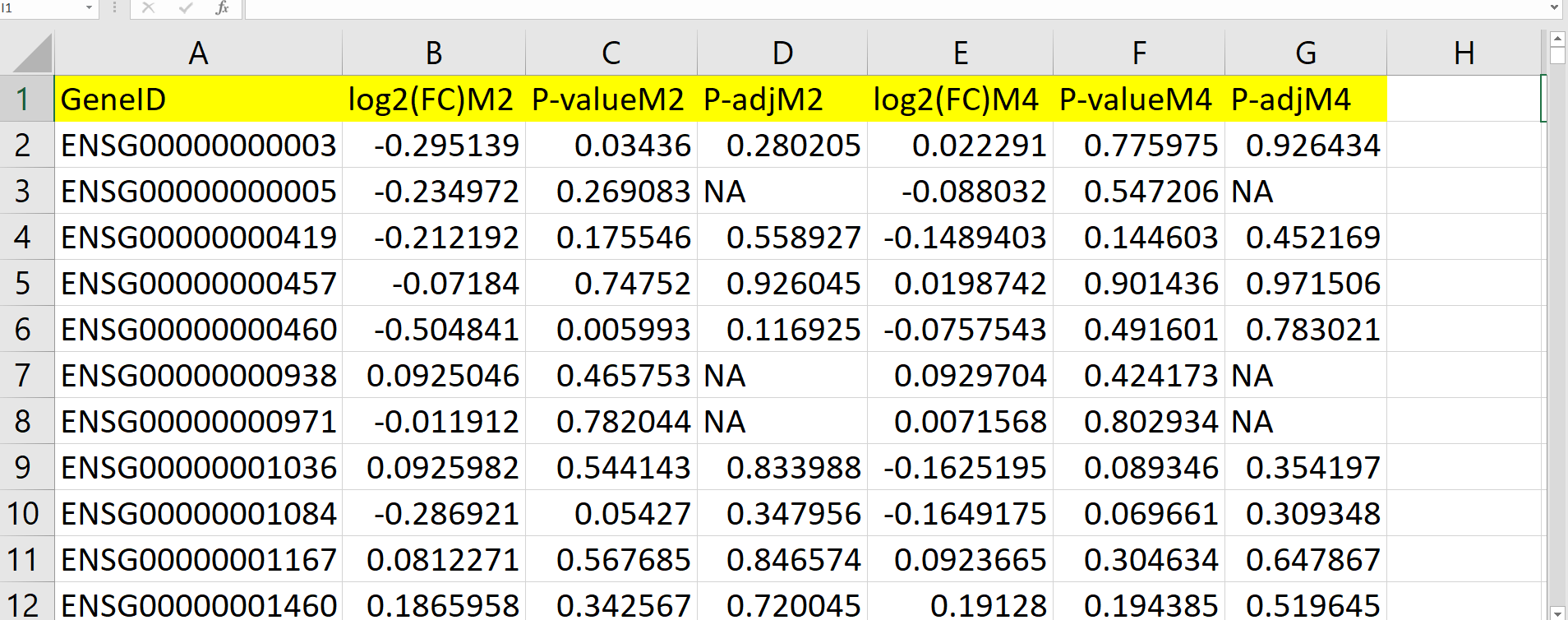

Supplement: Supplementary file 9 — Additional file 9. A-Lister source code. [file 12859_2019_3121_MOESM9_ESM.zip › A-Lister-master/Web/de_series_file.png]

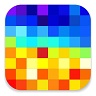

Supplement: Supplementary file 9 — Additional file 9. A-Lister source code. [file 12859_2019_3121_MOESM9_ESM.zip › A-Lister-master/Web/heat-map-icon.jpg]

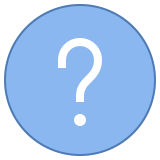

Supplement: Supplementary file 9 — Additional file 9. A-Lister source code. [file 12859_2019_3121_MOESM9_ESM.zip › A-Lister-master/Web/help.png]

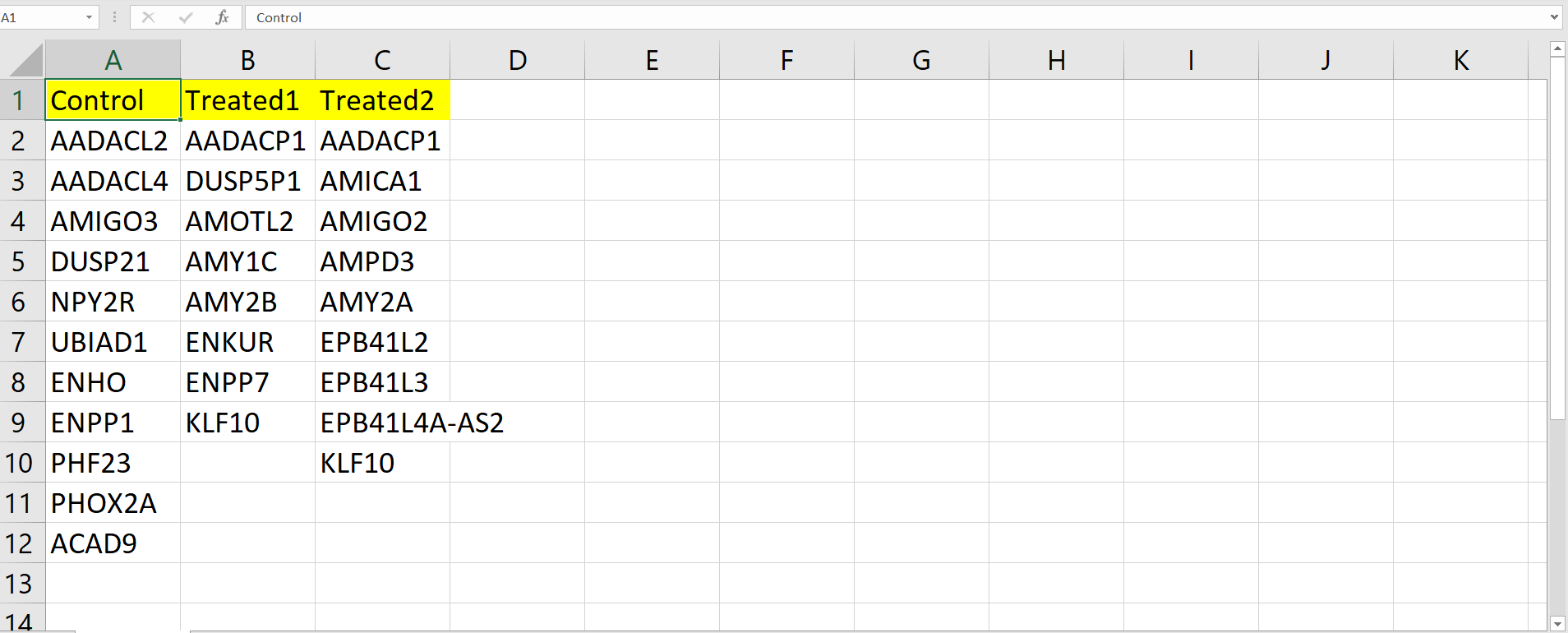

Supplement: Supplementary file 9 — Additional file 9. A-Lister source code. [file 12859_2019_3121_MOESM9_ESM.zip › A-Lister-master/Web/name_list_file.png]
